# Altered hepatic glucose homeostasis in AnxA6-KO mice fed a high-fat diet

**DOI:** 10.1371/journal.pone.0201310

**Published:** 2018-08-15

**Authors:** Rose Cairns, Alexander W. Fischer, Patricia Blanco-Munoz, Anna Alvarez-Guaita, Elsa Meneses-Salas, Antonia Egert, Christa Buechler, Andrew J. Hoy, Joerg Heeren, Carlos Enrich, Carles Rentero, Thomas Grewal

**Affiliations:** 1 School of Pharmacy, Faculty of Medicine and Health, University of Sydney, Sydney, NSW, Australia; 2 Department of Biochemistry and Molecular Biology II: Molecular Cell Biology, University Medical Center Hamburg-Eppendorf, Hamburg, Germany; 3 Departament de Biomedicina, Unitat de Biologia Cel·lular, Centre de Recerca Biomèdica CELLEX, Institut d’Investigacions Biomèdiques August Pi i Sunyer (IDIBAPS), Facultat de Medicina i Ciències de la Salut, Universitat de Barcelona, Barcelona, Spain; 4 Department of Internal Medicine I, Regensburg University Hospital, Regensburg, Germany; 5 Discipline of Physiology, School of Medical Science, Sydney Medical School, Charles Perkins Centre, University of Sydney, Sydney, NSW, Australia; University of Basque Country, SPAIN

## Abstract

Annexin A6 (AnxA6) controls cholesterol and membrane transport in endo- and exocytosis, and modulates triglyceride accumulation and storage. In addition, AnxA6 acts as a scaffolding protein for negative regulators of growth factor receptors and their effector pathways in many different cell types. Here we investigated the role of AnxA6 in the regulation of whole body lipid metabolism and insulin-regulated glucose homeostasis. Therefore, wildtype (WT) and AnxA6-knockout (KO) mice were fed a high-fat diet (HFD) for 17 weeks. During the course of HFD feeding, AnxA6-KO mice gained less weight compared to controls, which correlated with reduced adiposity. Systemic triglyceride and cholesterol levels of HFD-fed control and AnxA6-KO mice were comparable, with slightly elevated high density lipoprotein (HDL) and reduced triglyceride-rich lipoprotein (TRL) levels in AnxA6-KO mice. AnxA6-KO mice displayed a trend towards improved insulin sensitivity in oral glucose and insulin tolerance tests (OGTT, ITT), which correlated with increased insulin-inducible phosphorylation of protein kinase B (Akt) and ribosomal protein S6 kinase (S6) in liver extracts. However, HFD-fed AnxA6-KO mice failed to downregulate hepatic gluconeogenesis, despite similar insulin levels and insulin signaling activity, as well as expression profiles of insulin-sensitive transcription factors to controls. In addition, increased glycogen storage in livers of HFD- and chow-fed AnxA6-KO animals was observed. Together with an inability to reduce glucose production upon insulin exposure in AnxA6-depleted HuH7 hepatocytes, this implicates AnxA6 contributing to the fine-tuning of hepatic glucose metabolism with potential consequences for the systemic control of glucose in health and disease.

## Introduction

The liver is the central organ for many vital metabolic functions, including lipid and glucose homeostasis. This is exemplified in non-alcoholic fatty liver disease, which is characterized by excess hepatic lipid accumulation and closely linked to disorders with dysregulated glucose homeostasis, including type 2 diabetes, insulin resistance, obesity, and the metabolic syndrome [1,2]. Over the years, substantial progress has been made to dissect how abnormal lipid deposition in the liver interferes with the complex signaling networks that coordinate the balance of anabolic and catabolic pathways. Central to this intricate web of signals governing metabolic routes are a set of hormones, in particular insulin, modulating kinase and transcription factor activity, which then fine-tune expression, localization and action of numerous enzymes and transporters that control the level of critical metabolites in lipid and glucose metabolism [1–4].

In addition, regulatory proteins that control delivery of proteins and lipids to specific cellular destinations, enabling localized assembly and disassembly of multifactorial protein complexes in a spatiotemporal manner, are increasingly emerging as important control devices for liver function in health and disease [5–8]. This complex transport machinery includes Rab GTPases and soluble NSF attachment protein receptor proteins (SNAREs), as well as many others scaffolding and targeting proteins [5–8]. This also comprises the annexins, a group of twelve structurally related Ca^2+^- and phospholipid-binding proteins in humans, which contribute to regulate trafficking and signalling pathways through a variety of means [9,10]. Up to date, AnxA1, A2 and A7 have been associated with insulin sensitivity, glucose and lipid metabolism [11]. Diet-induced obese AnxA1-deficient animals showed increased adiposity, elevated glucose and insulin levels and development of insulin resistance [12]. On the other hand, AnxA1 or AnxA1-related peptides counteract non-alcoholic steatohepatitis [13], as well as atherosclerosis and cardiovascular disease triggered by hyperlipidemia [14–16]. AnxA2 interacts with insulin and insulin-like growth factor receptors [17], and is phosphorylated and sumoylated upon insulin stimulation [18,19], possibly relevant for cell adhesion [17], but also translocation of the glucose transporter type 4 (GLUT4) in adipocytes [20]. Moreover, AnxA2 regulates mouse extrahepatic low density lipoprotein receptor (LDLR) levels *in vivo* [21,22], which correlates with elevated LDL cholesterol levels in humans carrying AnxA2 gene variants [23]. Loss of AnxA7 was linked to abnormal insulin secretion [24]; however, these findings were challenged by others [25].

AnxA6 is the largest member of the annexin family and may also contribute to control whole body lipid and glucose metabolism. In adipocytes, we and others showed that AnxA6 is associated with lipid droplets [26–28], and AnxA6 depletion reduced lipolysis [28]. Together with increased AnxA6 levels in white adipose tissue from obese mice, this suggested AnxA6 levels to influence fatty acid release from adipocytes [28], a critical risk factor for the development of fatty liver disease. In hepatocytes, AnxA6 is highly abundant, making up approximately 0.25% of total liver protein [29,30]. Our laboratories found hepatic AnxA6 levels unchanged in murine non-alcoholic steatohepatitis and human liver fibrosis, but substantially reduced in hepatocellular carcinoma [31], which is linked to increased lipogenesis and cholesterol levels [32]. Other studies reported upregulation of AnxA6 levels in liver biopsies from obese humans and in women with type 2 diabetes [33, 34], Niemann-Pick Type C-deficient mice or mice fed a high-fat ketogenic diet [35, 36]. Hence, hepatic AnxA6 levels may be differentially regulated under metabolic stress.

Alike other annexins, AnxA6 is found at the plasma membrane, endosomes, but also secretory vesicles to organize membrane domains, cytoskeleton re-arrangements and signal complex formation [30,37–39]. As a putative regulator of hepatic lipid metabolism, AnxA6 modulates LDL endocytosis and LDL-cholesterol export from late endosomes [40,41]. In addition, we recently reported that AnxA6 co-localizes with lipid droplet markers in HuH7 hepatocytes [42]. Liver sections from AnxA6-KO mice displayed reduced lipid droplet numbers, which correlated with reduced triglyceride accumulation in AnxA6-deficient primary mouse hepatocytes and human HuH7 hepatocytes [42]. Furthermore, AnxA6 acts as a scaffold for p120 GTPase activating protein and protein kinase Cα, which are both negative effectors that downregulate epidermal growth factor receptor, Ras, mitogen activated protein kinase and Akt signaling pathways [37–39], all of which with multiple links to hepatic lipid and glucose homeostasis in normal and diseased settings [43–45].

In the present study, we assessed the influence of AnxA6 deficiency on lipid and glucose metabolism in HFD-fed mice. AnxA6-KO mice gained less weight compared to controls during the HFD feeding period, which correlated with reduced white adipose tissue mass. Lipoprotein profiles, kinetics of plasma glucose and insulin levels in oral glucose or insulin tolerance tests (OGTT, ITT), and magnitude of insulin-inducible Akt and S6 kinase phosphorylation suggest improved insulin sensitivity in HFD-fed AnxA6-KO animals. However, an inability to downregulate hepatic gluconeogenesis, despite normal insulin levels and insulin signaling, as well as expression profiles of insulin-sensitive transcription factors, indicate that AnxA6 deficiency compromises regulatory steps in hepatic glucose homeostasis that only become apparent during metabolic stress. The potential consequences for the systemic control of glucose in health and disease are discussed.

## Materials and methods

### Reagents and antibodies

DMEM, geneticin (G418), puromycin, pyruvate, oleic acid, β-D(+)-glucose, and paraformaldehyde were from Sigma. Insulin (Actrapid®) was from Novo Nordisk (Bagsværd, Denmark). ELISA kits for adiponectin (DuoSet® ELISA mouse adiponectin/Acrp30) and leptin (DuoSet® ELISA mouse Leptin) were from R&D Systems (Minneapolis, MN, USA). The Ultra-Sensitive Rat Insulin ELISA Kit was obtained from Crystal Chem (Downers Grove, IL, USA). SDS-PAGE molecular weight markers were from Fermentas. Polyclonal rabbit anti-AnxA6 was prepared in our laboratories [40,46]. Rabbit polyclonal antibodies against total and phosphorylated S6, Akt, insulin receptor substrate 1 (IRS1), mechanistic target of rapamycin (mTOR), 5' adenosine monophosphate-activated protein kinase α (AMPKα), extracellular signal-regulated kinase (Erk1/2), as well as Forkhead box O1 (FoxO1), liver X receptor (LXR), AnxA2, glyceraldehyde 3-phosphate dehydrogenase (GAPDH), horseradish peroxidase (HRP)-labeled secondary goat anti-mouse and goat anti-rabbit antibodies, were from Cell Signaling. Rabbit antibodies against peroxisome proliferator-activated receptor α (PPARα), and sterol regulatory element-binding protein 1 (SREBP1) were from Santa Cruz. Rabbit anti-insulin receptor β antibody was from Upstate, rabbit anti-low density lipoprotein receptor-related protein 1 antibody was from Epitomics, rabbit anti-apolipoprotein E antibody was from Acris, and goat anti-LDLR antibody was from R&D Systems. Rabbit polyclonal anti-β-actin and anti-scavenger receptor BI (SR-BI) antibodies were from BD Transduction Laboratories and Novus, respectively. HuH7 cells were from the American Type Culture Collection (ATCC, Manassas, VA, USA).

### Animals

Eight weeks old male WT and AnxA6-KO C57BL/6J mice (n = 25-27/group) [47] were held single caged at 23°C in a 12:12 h light-dark cycle, allowed access to water and a high fat diet *ad libitum* (Bio-Serv F3282, High Fat Diet 35.5% lard; Frenchtown, NJ, USA) for 17 weeks. Eight to twelve weeks old WT and AnxA6-KO animals (n = 4) on a chow diet were analyzed in some experiments. Every effort was made to minimize animal suffering and to use the minimum number of animals per group and experiment. Bodyweight, food intake and core body temperature were monitored regularly. All animal care and experimental procedures were approved by the Animal Welfare Officers of University Medical Center Hamburg-Eppendorf, the Behörde für Soziales, Familie, Gesundheit und Verbraucherschutz (Hamburg, Germany) and the local ethics committee at the University of Barcelona following European (2010/63/UE) and Spanish (RD53/2013) regulations for the care and use of laboratory animals.

### Determination of hormones and lipids from blood and liver tissue

For the determination of triglycerides and cholesterol in plasma and lipoproteins (n = 3), leptin and adiponectin (n = 14), animals were fasted for 4 h. Blood samples were taken and centrifuged for 5 min at 10,000 rpm and the supernatant (plasma) was used for hormone and lipid measurements. ELISAs to determine serum concentrations of insulin, leptin and adiponectin were performed according to manufacturer’s instructions [48]. Total serum triglycerides and cholesterol levels were quantified using commercial kits (Roche) according to manufacturer’s instructions [49]. For lipoprotein profiling, pooled plasma samples from 3 mice per genotype were separated into TRL, HDL and glycerol by fast-performance liquid chromatography (FPLC) into 40 fractions using S6-superose columns (GE Healthcare, Piscataway, NJ Cleveland, OH, USA). Triglyceride, glycerol and cholesterol levels in each fraction as well as liver samples (see below) were determined using commercial kits as described above [49].

For the determination of hepatic phospholipid content, 50–100 mg liver tissue (n = 4 per group) was homogenized and samples were analyzed by mass spectrometry as described previously [50]. In brief, liver samples containing 650 μg protein were mixed with an internal standard containing 100 μM phosphatidylcholine (19:0/19:0), phosphatidylethanolamine (17:0/17:0), phosphatidylserine (17:0/17:0), phosphatidylglycerol (17:0/17:0) and phosphatidic acid (17:0/17:0) (all from Avanti Lipids, Alabaster, AL, USA) as described [50]. After lipid extraction, samples were diluted 10–100-fold in 2:1 methanol:chloroform with 5 mM ammonium acetate and mass spectrometry data was acquired on a hybrid triple quadrupole linear ion trap mass spectrometer (QTRAP 5500 AB Sciex, Framingham, MA, USA) equipped with an automated chip-based nano-ESI source (TriVersa Nanomate, Advion Biosciences, Ithaca, NY, USA). Lipids were identified and quantified using LipidView 1.3beta (AB Sciex) software after correction for isotope contributions.

### *In vivo* insulin response

For the determination of insulin response, animals were fasted for 4 h to receive insulin (1.5 U/kg bodyweight) or saline via intraperitoneal injection (n = 2 per group). Blood glucose was measured at 0 and 15 min post-injection (see details below). Mice were then sacrificed by cervical dislocation, livers were removed and snap frozen in liquid nitrogen. Membrane, cytosolic and nuclear fractions were prepared for western blot analysis (see below).

### Oral glucose tolerance test (OGTT)

Animals were starved for 4 h prior to administration of 1 g β-D (+)-glucose/kg bodyweight via oral gavage (n = 25–27) [49]. Glucose was determined in blood obtained from a small incision in the mouse tail and pressed on a glucometer strip at 0, 15, 30, 60, 90 and 120 min post-gavage. Blood glucose levels were measured using the Accu-Check® Aviva blood glucose monitor (Roche) [48]. Plasma insulin was measured using an ELISA at 0 and 15 min post-gavage in 4–5 mice per genotype.

### Pyruvate tolerance test (PTT)

Animals were starved for 4 h prior to administration of pyruvate (2 g/kg bodyweight) via intra-peritoneal injection [51]. Blood glucose and insulin levels at multiple time points over a 2–4 h period were measured as described above (n = 5–19 per time point). Mice were then anaesthetized at the different time points after pyruvate injection using ketamin-xylazine injection anesthesia and transcardially perfused with ice-cold PBS. Livers were removed and snap frozen in liquid nitrogen. Membrane, cytosolic and nuclear fractions were prepared for western blot analysis (n = 4 per group at t = 0 min and 120 min after pyruvate injection; see below).

### Preparation of membrane and cytosolic fractions from mouse liver

Mouse liver tissue (n = 2–4 per group) was snap frozen in liquid nitrogen immediately following sacrifice. Tissue samples (200 mg) were homogenized in 20 mM Tris-HCl pH 7.4, 2 mM MgCl_2_, 0.25 M sucrose (with protease inhibitor cocktail; Roche, Basel, Switzerland) using a TissueLyser (Qiagen). The sample was centrifuged at 800 g for 15 min at 4°C, and the supernatant was cleared using the same conditions. For membrane and cytosol separation, the supernatant was then centrifuged at 100,000 g for 1 h at 4°C. The supernatant (cytosol) was removed, and the membrane pellet was resuspended in 50 mM Tris-HCl pH 8.0, 2 mM CaCl_2_, 80 mM NaCl, 1% (v/v) Triton X-100, 0.1% (w/v) SDS, and protease inhibitors. Samples were again centrifuged at 100,000 g for 30 min at 4°C to separate nuclei (pellet) from cellular membranes (supernatant). The protein content of the membrane, nuclear and cytosolic fractions was determined [52], and samples were analyzed by western blotting (see below).

For the preparation of extracts from HuH7 hepatocytes, cells were harvested in lysis buffer (20 mM Tris-HCl, pH 7.5, 2 mM EDTA, 100 mM NaCl, 5 mM MgCl_2_, 1% (v/v) Triton X-100, 5 mM NaF, 10% (v/v) glycerol, 0.5% (v/v) 2-mercaptoethanol, 0.1 mM Na_3_VO_4_ and protease inhibitors). After centrifugation at 10,000 g the protein concentration of the cleared cell lysate was determined. Cell lysates were separated by 10 or 12.5% SDS-PAGE and transferred to Immobilon-P (Millipore) for western blot analysis (see below).

### RNA extraction from mouse liver and real-time quantitative PCR

Liver samples (100 mg) were homogenized in 1 ml TRIzol® (Invitrogen) in a TissueLyser (Qiagen) at 20 Hz for 6 min (n = 4 per group at t = 0 min and 120 min after pyruvate injection). 200 μl chloroform was added, samples were vortexed and centrifuged for 15 min at 14,000 g. The supernatant was mixed with 100% ethanol (1/3 v/v) and RNA was extracted using the RNA extraction kit (NucleoSpin® RNA kit, Macherey-Nagel) according to the manufacturer’s instructions. RNA purity and concentration was determined using the NanoDrop ND-1000 (PeqLab). cDNA synthesis was performed with 400 ng RNA using the High Capacity cDNA Reverse Transcription Kit (Applied Biosystems) with random hexamer primers following the manufacturer's instructions. Relative mRNA levels were determined by semiquantitative RT-PCR (qRT-PCR) using the TaqMan Gene Expression Assay Technique and the 7900HT Fast Real-Time PCR System (Applied Biosystems). The following TaqManAssay-on-Demand primer sets for mouse gene expression analysis were purchased from Invitrogen: AnxA6 (*mAnxA6*, Mm00478966_m1), carnitine palmitoyltransferase 1A (CPT1α; *mCpt1a* liver, Mm00550438_m1), fatty acid synthase (*mFasn*, Mm00662319_m1), fibroblast growth factor 21 (Fgf21; *mFgf21*, Mm00840165_g1), glucose-6 phosphatase (G6P) catalytic (*mG6pc*, Mm00839363_m1), solute carrier family 2 (facilitated glucose transporter), member 2 (SLC2A2, GLUT2; *mSlc2a2*, *mGlut2*, Mm00446224_m1), insulin induced gene 1 (Insig1; *mInsig1*, Mm00463389_m1), hydroxyacyl-Coenzyme A dehydrogenase/3-ketoacyl-Coenzyme A thiolase/enoyl-Coenzyme A hydratase (trifunctional protein), α (*mHadha*, Mm00805228_m1) and β subunit (*mHadhb*, Mm00523880_g1, LDLR (*mLdlr*, Mm00440169_m1), pyruvate dehydrogenase lipoamide kinase isozyme 4 (*mPdk4*, Mm00443325_m1), TATA-box binding protein (Tbp; *mTbp* (Mm00446973_m1). Gene of interest cycle threshold (Ct) values were normalized to the housekeeper Tbp mRNA levels, using the ΔΔCT method [53].

Relative mRNA levels in HuH7 hepatocytes were determined by quantitative real-time PCR in triplicates using SYBR-Green and the 7900HT Fast Real-time PCR System (Applied Biosystems) as described previously [42]. In brief, total RNA was isolated and cDNA synthesis was performed with 500 ng of RNA using the High Capacity cDNA Reverse Transcription Kit (Applied Biosystems) with random hexamer primers (AnxA1: forward 5’-AATCCATCCTCGGATGTCGC-3’, nt 335–354; reverse 5’-TCAGTGTTTCATCCAGGGGC-3’, nt 478–497; AnxA6: forward 5’-TGGCTGCTGAGATCTTGGAAA-3’, nt 1679–1699; reverse 5’-GATCGTCATGAAACGTGTCTCC-3’, nt 1734–1755; PDK4: forward 5’-TCAGACAGAGGAGGTGGTGTT-3’, nt 1194–2114; reverse 5’-AAAACCAGCCAAAGGAGCATTC, nt 2189–3110; 28s rRNA: forward 5’-GTTGTTGCCATGGTAATCCTGCTCAGTAC-3’, nt 4535–4564, reverse 5’-TCTGACTTAGAGGCGTTCAGTCATAATCC-3’, nt 4638–4667) following the manufacturer's instructions. Gene of interest cycle threshold (Ct) values were normalized to 28s rRNA housekeeper levels (ΔΔCt method) as described [42].

### SDS-PAGE and western blotting

Membrane and cytosol fractions from mouse liver tissue (n = 2–4 per group) were separated by 10 or 12% SDS-PAGE (NuPAGE® Novex® 4–12% Bis-Tris Protein Gels, Invitrogen) and transferred to nitrocellulose (Whatman) using a MiniTrans-Blot® cell module (BioRad). Proteins were detected using their specific primary antibodies, followed by HRP-conjugated secondary antibodies and enhanced chemiluminescence detection (ECL, Amersham). For each sample and protein of interest, at least 2 individual blots were performed. The intensity of bands was quantified using Image Studio Lite Ver. 3.1 (LI-COR) and results were normalized to β-actin. Relative levels of phosphorylated Akt, Erk1/2, mTOR, S6 and IRS1 were normalized to total Akt, Erk1/2, mTOR, S6 and IRS1, respectively.

For western blot analysis of AnxA6 and GAPDH expression in HuH7 hepatocytes, cell lysates were separated by 10 or 12.5% SDS-PAGE and transferred to Immobilon-P (Millipore). AnxA6 and GAPDH proteins were detected using their specific primary antibodies, followed by HRP-conjugated secondary antibodies and enhanced chemiluminescence detection (ECL, Amersham).

### Haematoxylin and eosin (H&E) staining of liver sections

For histological analysis, mice were anaesthetised as above and perfused with 4% (w/v) paraformaldehyde and 5% (w/v) sucrose. A piece of liver was fixed for paraffin embedding and H&E staining was performed as described (n = 4 per group) [54].

### Electron microscopy of mouse liver sections

For transmission electron microscopy (TEM), liver tissue was isolated from mice (n = 4 per group) following anaesthesia after intracardial perfusion with 2.5% glutaraldehyde in phosphate buffer. Liver samples were then fixed overnight in 2.5% glutaraldehyde and 4% paraformaldehyde. Small cubes of 1 mm^3^ were post-fixed in osmium tetroxide and embedded in Spurr (Sigma Aldrich) [42,55]. TEM images were acquired from ultrathin sections using a JEOL-1010 electron microscope (JEOL USA, Peabody, MA, USA) with a SC1000 ORIUS-CCD digital camera (Gatan, Pleasanton, CA, USA).

### Glycogen and production of glucose and ketone bodies in primary hepatocytes

Primary hepatocytes from WT and AnxA6 KO-mice were isolated (n = 5 per group) as described previously [42]. To determine gluconeogenic capacity, 2.5 x 10^5^ primary hepatocytes were seeded in triplicates and grown overnight in DMEM, 2% FCS, 1 mM methionine and 200 nM dexamethasone, fasted for 6 h without glucose, and then incubated with 2 mM pyruvate or 20 mM glycerol for 24 h. Media was stored for glucose measurement. For cellular glycogen quantification, cells were lysed in 180 μl 1 M KOH, equilibrated with 45 μl Na_2_SO_4_, and glycogen was precipitated with 0.975 ml 100% ethanol, followed by centrifugation at 15,000 g for 15 min at 4°C. The pellet was resuspended in 110 μl deionized H_2_O, and glycogen was precipitated with 0.990 ml 100% ethanol. After centrifugation at 15,000 g for 15 min at 4°C, the precipitated glycogen was digested to glucose with 100 μl amyloglucosidase (Sigma-Aldrich; 3 mg in 10 ml 0.25 M acetate buffer) at 37°C for 2 h [56]. Media glucose and glycogen-derived glucosyl levels were determined using the Glucose GO assay kit (Sigma).

For ketone bodies, cells were grown in DMEM, 2% FCS, 1 mM methionine and 200 nM dexamethasone (Sigma) overnight. 2-hydroxybutyrate levels in the media were measured using a colorimetric assay following manufacturer’s instructions (Cayman Chemical).

### Glycogen and glucose production in HuH7 hepatocytes

Wildtype (HuH7-WT) and AnxA6-depleted HuH7 hepatocytes (HuH7-A6KD) were grown in DMEM, with 10% fetal calf serum, L-glutamine (2 mM), penicillin (100 U/ml) and streptomycin (100 μg/ml) at 37°C, 5% CO_2_. For the generation of HuH7-A6KD, 1–2 × 10^6^ cells were transfected with 1.5 μg SureSilencing shRNA plasmid (SABiosciences) targeting human AnxA6 at position 352–372 (5′-gcaaggacctcattgctgatt-3′) and Lipofectamine 2000 according to the manufacturer's instructions. After 48 h, cells were selected in the presence of 100 μg/ml puromycin. After 2 weeks, puromycin-resistant and AnxA6-depleted colonies were identified [57]. Cells were seeded in triplicate onto six-well plates in Krebs buffer and treated ± 2 mM pyruvate and ± 100 nM insulin for 2 h. Cells were washed with ice-cold PBS and lysed in 300 μl 1 M KOH. For glycogen extraction, 75 μl saturated Na_2_SO_4_ and 1.625 ml 100% ethanol was added and the sample was centrifuged at 15,000 g for 15 min at 4°C. The pellet was resuspended in 200 μl deionized H_2_O, and glycogen was precipitated with 1.8 ml 95% ethanol. The sample was centrifuged at 15,000 g for 15 min at 4°C and the precipitated glycogen was digested to glucose with 250 μl amyloglucosidase at 37°C for 2 h [56]. Media glucose and glycogen-derived glucosyl levels were determined using the hexokinase reagent (GAHK20, Sigma-Aldrich) according to manufacturer’s instructions.

### Statistics

Statistical analysis was carried out using Microsoft Excel and GraphPad Prism 6. Data represent means ± SEM of at least three independent experiments unless otherwise stated. Statistical comparison was performed using an unpaired Student’s *t-*tests or two-way ANOVA test (* = p < 0.05, ** = p < 0.01, *** = p < 0.001).

## Results

### AnxA6-KO animals display normal liver functions under standard growth conditions

**Fig 1:**
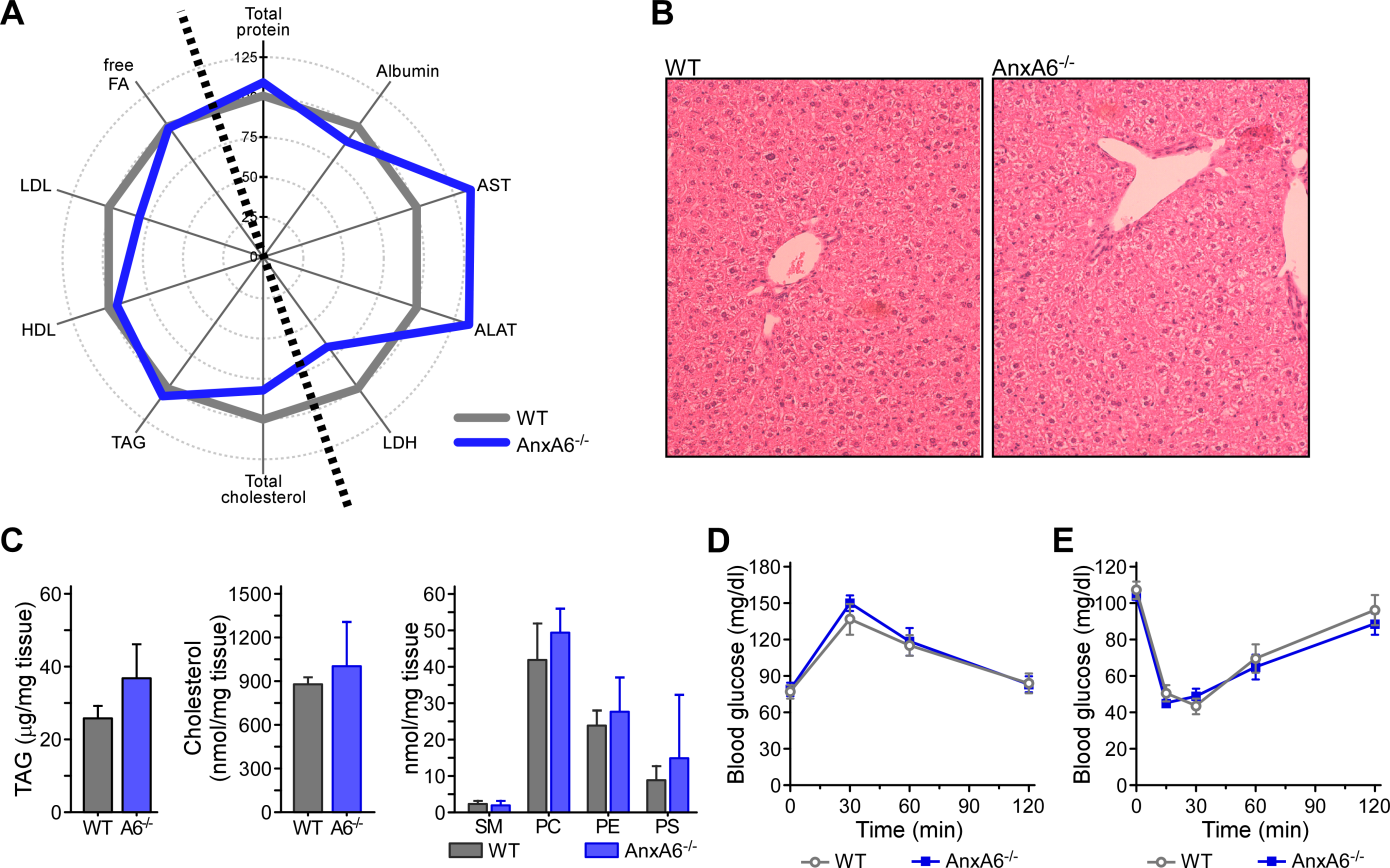
Liver function, lipid and insulin-regulated glucose metabolism are normal in chow-fed AnxA6-KO mice. **(A)** Spider diagram presentation of total protein, albumin, aspartate aminotransferase (AST), alanine aminotransferase (ALAT), lactate dehydrogenase (LDH), as well as total cholesterol, triglycerides (TAG), low density lipoprotein (LDL), high density lipoprotein (HDL), and free fatty acids (free FA) in the plasma of 8–12 weeks old wildtype (WT) and AnxA6-KO animals (AnxA6^-/-^) on a chow diet (n = 4). The data are presented relative to WT (100%). For absolute values, see text and [28] for further details. **(B)** H&E staining of liver sections from WT and AnxA6-KO mice on a chow diet. **(C)** Liver homogenates from 8–12 weeks old WT and AnxA6-KO mice were analyzed for triglyceride, cholesterol and phospholipid content as described in Material and Methods (mean ± SEM, n = 8). SM, sphingomyelin; PC, phosphatidylcholine; PE, phosphatidylethanolamine; PS, phosphatidylserine. **(D)** WT and AnxA6-KO mice on a chow diet were starved for 4 h prior to receive glucose via oral gavage (1 g/kg body weight). Blood glucose levels were determined 0–120 min after glucose administration (mean ± SEM, n = 6). **(E)** WT and AnxA6-KO mice received insulin (1.5 U/kg bodyweight) or saline via intraperitoneal injection. Blood glucose was measured at t = 0–120 min post-injection (mean ± SEM, n = 6). **(D-E)** Data points for t = 0 and 30 min have been described previously [28].

We recently identified reduced triglyceride accumulation in primary AnxA6-deficient hepatocytes upon oleate supply and lower lipid droplet numbers in liver sections from AnxA6-KO mice [42], indicating AnxA6 to participate in hepatic metabolism and liver physiology. In line with previous studies [28,47], plasma levels of the liver enzymes aspartate aminotransferase (67 ± 10.6 in WT vs. 90.5 ± 22.1 U/l in AnxA6-KO), alanine aminotransferase (25 ± 9.9 vs. 33.5 ± 5.1 U/l) and lactate dehydrogenase (858 ± 268.2 vs. 583 ± 164.1 U/l), as well as albumin (26.5 ± 1 vs. 23.5 ± 1.7 g/l) and total plasma protein (42.5 ± 4.9 vs. 46 ± 4.1 g/l) (Fig 1A) indicated normal liver function in AnxA6-KO animals fed a standard chow diet. Furthermore, body weight (29 ± 2 vs. 29 ± 9 g), total plasma cholesterol (82 ± 3 vs. 65 ± 8 mg/dl), triglycerides (70 ± 15 vs. 80 ± 5 mg/dl), free fatty acids (1.6 ± 0.3 vs. 1.5 ± 0.2 μmol/l), LDL (32 ± 2 vs. 24 ± 4 mg/dl), and HDL (38 ± 2 vs. 33 ± 3 mg/dl) in 8–12 weeks old control and AnxA6-KO animals were alike (Fig 1A). Likewise, H&E staining of liver sections (Fig 1B) and triglyceride, cholesterol and phospholipid content in liver tissue supported normal liver morphology and hepatic lipid metabolism in mice lacking AnxA6 (Fig 1C). Also, fasting blood glucose measured prior to OGTTs were similar in both genotypes (75 ± 5 mg/dl), as was the handling of the glucose challenge (140 ± 10 mg/dl at t = 30 min; Fig 1D). Likewise, glucose levels in WT and AnxA6-KO mice were similar (100 ± 15 mg/dl) and decreased 30 min after insulin injection (45 ± 5 mg/dl) to return to baseline thereafter (Fig 1E). Hence, under normal (chow diet) conditions, overall liver function, lipid and insulin-regulated glucose metabolism in AnxA6-KO mice appeared similar to control animals.

### Less weight gain of AnxA6-KO mice after HFD feeding

**Fig 2:**
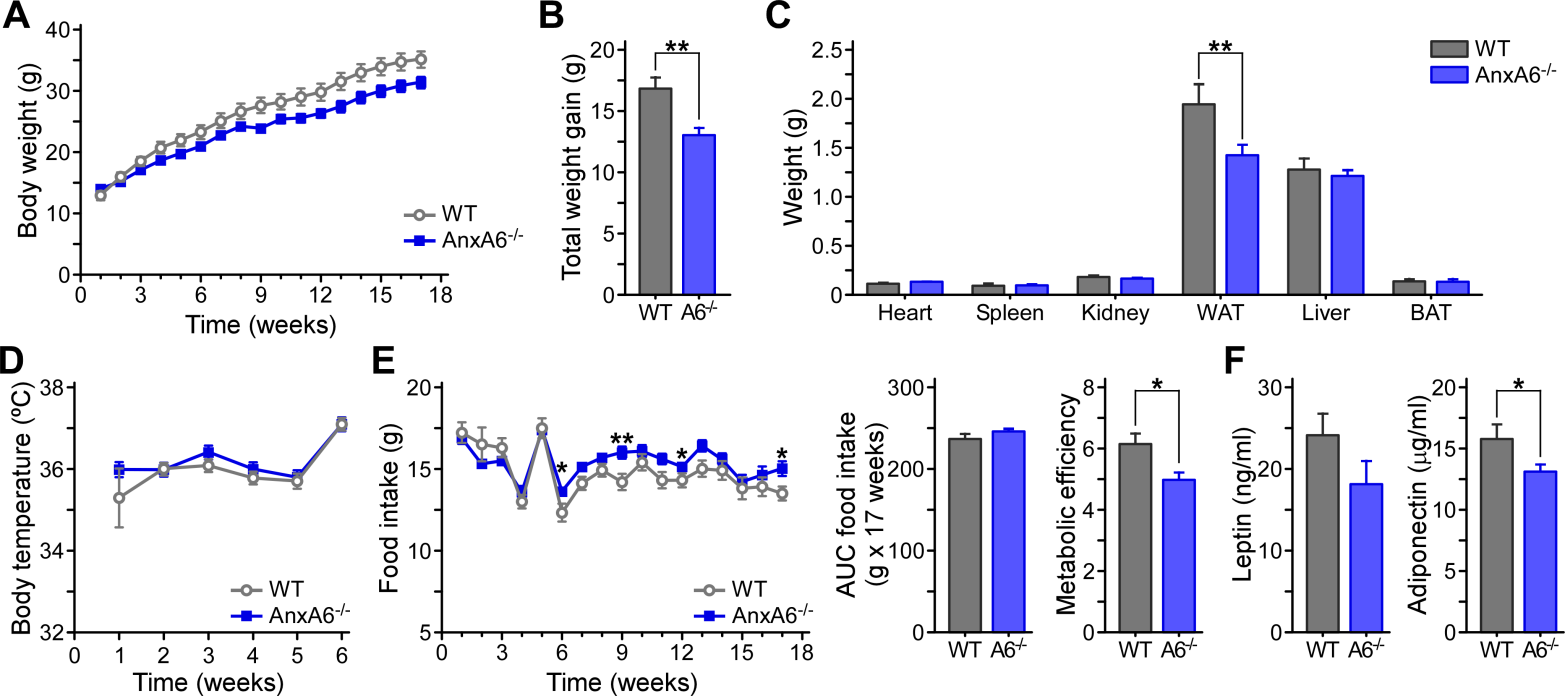
Reduced weight gain and less adiposity of AnxA6-KO mice after HFD feeding. 8 weeks old male WT and AnxA6-KO (AnxA6^-/-^, A6^-/-^) C57BL/6J mice were fed a high fat diet (HFD) for 17 weeks. **(A)** Weekly weight gain over the 17 week HFD feeding period. **(B)** Cumulative weight gain after 17 week HFD feeding (mean ± SEM, n = 25–27). **(C)** Organ weight comparison after 17 week HFD feeding. AnxA6-KO mice had significantly less white adipose tissue (WAT; epididymal + subcutaneous WAT) than WT mice (mean ± SEM, n = 7–8 for heart, spleen, kidney, BAT; n = 14 for WAT; n = 22 for liver). Brown adipose tissue (BAT). **(D)** Body temperature over 6 weeks HFD feeding (mean ± SEM, n = 14). **(E)** Food intake over the HFD feeding period. AnxA6-KO mice consumed slightly more in week 6, 9, 12, and 17, but the area under the curve (AUC) of total food intake over the 17 week HFD feeding period was comparable. The metabolic efficiency (% food stored as body weight) was reduced in AnxA6-KO mice (mean ± SEM, n = 14). **(F)** Serum from HFD-fed WT and AnxA6-KO mice was analyzed for leptin and adiponectin levels using ELISA as described (mean ± SEM, n = 14). ** P < 0.01, * P <0.05 (Student’s T-test).

To examine if physiological stress mimicking conditions of constant overfeeding could reveal novel roles for AnxA6 in lipid and glucose homeostasis, we studied the metabolic phenotype of AnxA6-KO mice after 17 weeks of HFD feeding. These conditions are well known to induce metabolic stress, hepatic lipid accumulation, obesity and onset of insulin resistance in C57BL/6 mice [58]. We first monitored whole body and organ weight, food intake and body temperature over the HFD feeding period (Fig 2). HFD feeding was associated with weight gain in both mouse strains (Fig 2A). However, HFD-fed AnxA6-KO mice gained less weight over the feeding period, with average total weight gain 22.6% lower compared to control mice (Fig 2B; WT 16.8 ± 0.9 g, AnxA6-KO 13.0 ± 0.61 g; ** p < 0.01). After 17 weeks HFD, the weight of the heart, spleen, kidney, liver and brown adipose tissue were comparable in both strains (Fig 2C). In line with reduced body weight, white adipose tissue mass was significantly reduced in AnxA6-KO mice (WT 1.94 ± 0.202 g, AnxA6-KO 1.432 ± 0.0971 g; ** p <0.01). The difference in adiposity was not associated with body temperature (Fig 2D) or food intake (Fig 2E), thereby excluding increased energy expenditure through thermogenesis or reduced nutrient uptake as a cause for reduced weight gain in the HFD-fed AnxA6-KO mice. Interestingly, metabolic efficiency (percentage of ingested food stored as body weight), which is an indirect marker of intestinal uptake capacity and energy expenditure [59], was significantly lower in AnxA6-KO mice, indicating reduced food absorption in the gut or increased energy expenditure (Fig 2E). Serum leptin and adiponectin levels in WT and AnxA6-KO mice (Fig 2F) were within the expected range following HFD feeding [60]. The trend of reduced leptin levels observed in AnxA6-KO mice could be due to the decrease in adipose tissue mass, possibly lowering the net amount of secreted adipokines. Taken together, the overall reduction in HFD-induced weight gain in the AnxA6-KO mice correlated with reduced adiposity, which may reflect the recently observed impaired proliferation in AnxA6-depleted pre-adipocytes [28], or alternatively, altered metabolic efficiency [59].

### Plasma lipid profiling in HFD-fed AnxA6-KO mice

**Fig 3:**
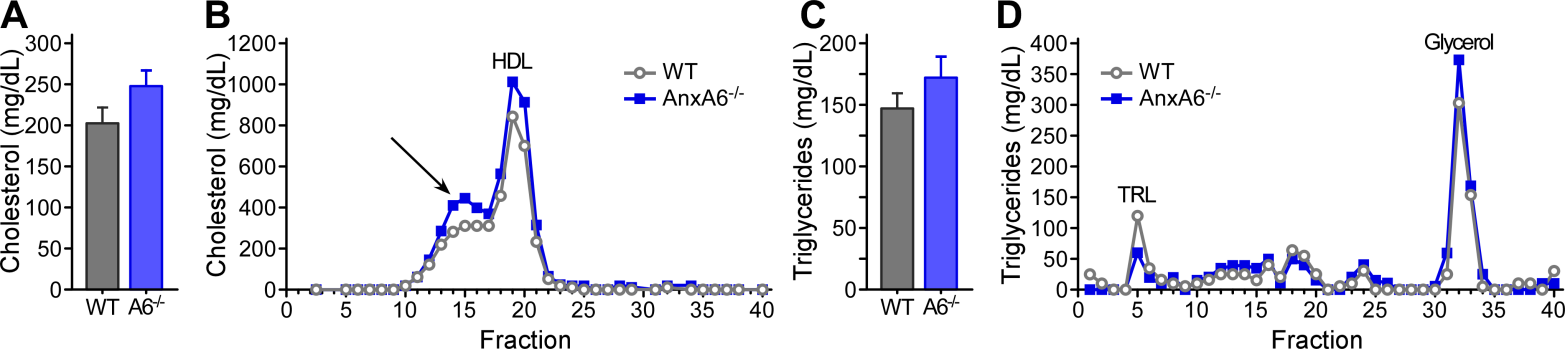
Serum triglyceride and cholesterol profiling in HFD-fed WT and AnxA6-KO mice. **(A, C)** Total cholesterol and triglyceride levels showed no significant differences between genotypes. **(B, D)** Pooled serum samples from 3 mice of each genotype were separated by fast-performance liquid chromatography (FPLC) and each fraction was analyzed for cholesterol and triglyceride content. **(B)** Cholesterol profiling with HDL (fractions #18–20) and neighboring cholesterol-rich fractions (see arrow; #13–17), likely representing HDL- or LDL subspecies. **(D)** Triglyceride-rich lipoproteins (TRL, fraction #5) and free glycerol (fractions #31–34).

Prolonged HFD feeding induces dyslipidemia [58] and we next compared lipid levels and lipoprotein profiles in the plasma of WT and AnxA6-KO mice. Total plasma cholesterol levels were similar in HFD-fed WT (203.57 ± 20 mg/dl) and AnxA6-KO animals (249.25 ± 20 mg/dl) (Fig 3A), reflecting an approximately 2.5–4 -fold increase compared to their chow-fed controls (see Fig 1A). Similarly, total plasma triglycerides in HFD-fed WT and AnxA6-KO animals were comparable (Fig 3C) and elevated approximately 2–2.5 -fold (148.16 ± 10 mg/dl in WT; 173.44 ± 10 mg/dl in AnxA6-KO) compared to their chow-fed counterparts (see Fig 1A).

To compare the lipoprotein profiles of HDF-fed WT and AnxA6-KO mice, we next carried out FPLC analysis. Animals were fasted for 4 h and pooled serum samples from three HFD-fed mice of each genotype were separated by FPLC and each fraction analyzed for cholesterol and triglyceride content as described [49]. Cholesterol profiling (Fig 3B) in HFD-fed WT mice showed HDL as the prominent peak (fractions #18–20), with neighboring cholesterol-rich fractions (#13–17) likely representing HDL- or LDL subspecies [61]. AnxA6-KO mice showed a similar pattern, however cholesterol levels in the fractions containing HDL and other cholesterol-rich lipoproteins were slightly increased (Fig 3B). On the other hand, triglyceride-rich lipoproteins (fraction #5) were reduced in HFD-fed AnxA6-KO mice compared to WT mice (Fig 3D). Glycerol levels (fractions #31–34) in HFD-fed AnxA6-KO mice were comparable to those from WT mice, indicating lipolysis being functional in adipose tissue of AnxA6-KO mice. These subtle differences in lipoprotein profiles were not associated with altered expression of hepatic lipoprotein receptors (LDLR, SR-BI, low density receptor-related protein 1) and apolipoprotein E (S1A, S1B, S1C and S2 Figs), which facilitates cellular uptake of TRL [62]. However, as insulin lowers the production of hepatic triglyceride-rich lipoproteins [63] and increases lipolysis of plasma TRL [64], yet improves HDL levels and function [65], the lipoprotein profiles of AnxA6-KO mice could indicate increased impact of insulin on lipid metabolism in these mice.

### Increased insulin signaling in livers from HFD-fed AnxA6-KO mice

**Fig 4:**
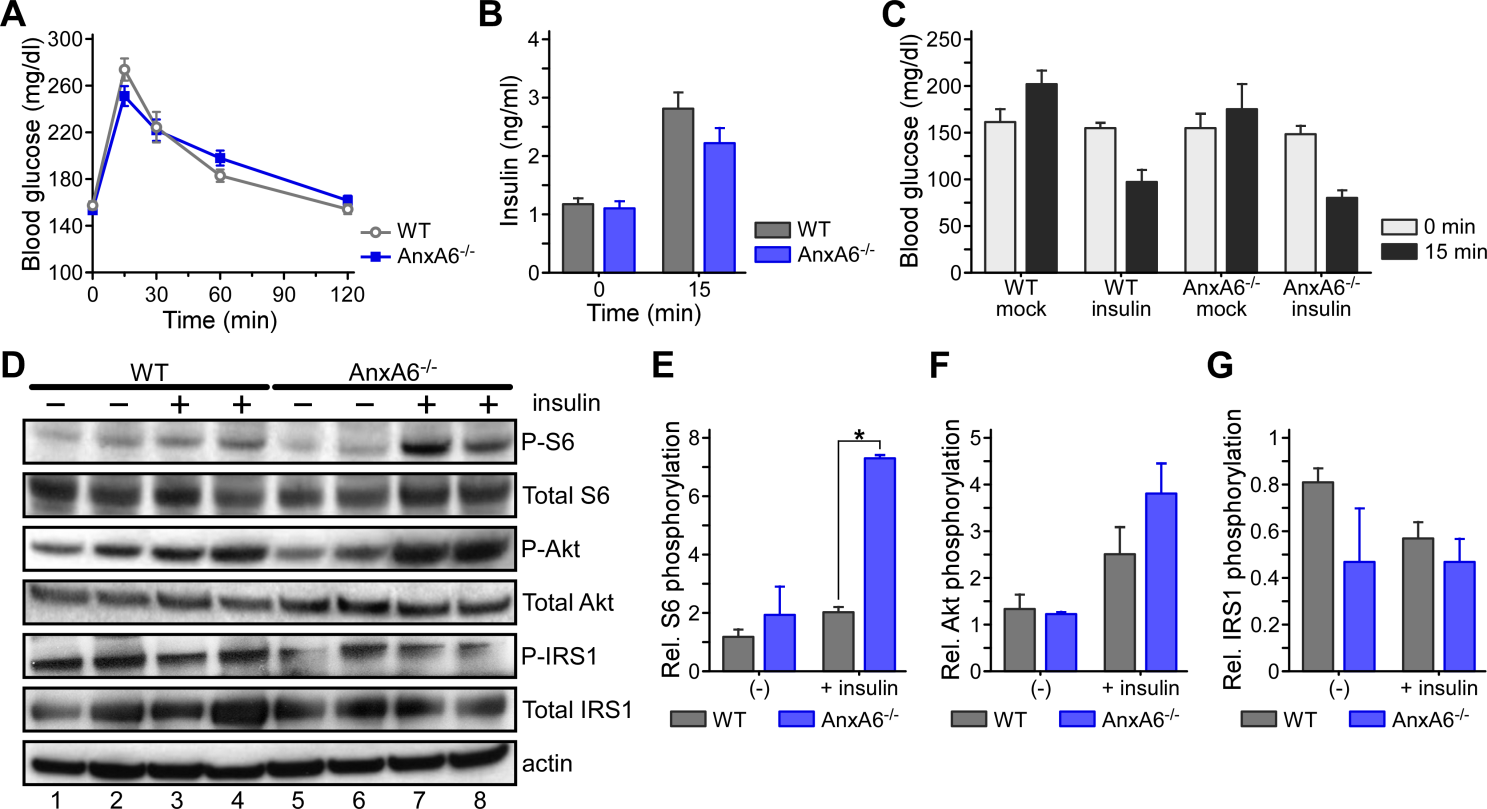
Glucose and insulin tolerance tests (OGTT, ITT) and insulin signaling in HFD-fed WT and AnxA6-KO mice. **(A, B)** HFD-fed WT and AnxA6-KO (AnxA6^-/-^) mice were starved for 4 h prior to receive glucose via oral gavage (1 g/kg body weight). **(A)** Blood glucose levels were determined 0–120 min after glucose administration (mean ± SEM, n = 25–27). **(B)** Plasma insulin levels before and 15 min after oral glucose gavage (mean ± SEM, n = 14). **(C-D)** HFD-fed WT and AnxA6-KO animals (2 per group) were fasted for 4 h to receive insulin (1.5 U/kg bodyweight) or saline via intraperitoneal injection. **(C)** Blood glucose was measured at t = 0 min and t = 15 min post-injection. **(D)** Livers were removed and cytosolic fractions were analyzed by western blotting for phosphorylated and total ribosomal S6 kinase (P-/total S6), Akt kinase (P-/total Akt), insulin receptor substrate 1 (P-/total IRS1) and β-actin. **(E-G)** Relative levels of activated S6, Akt and IRS1 were quantified and normalized to total S6, Akt and IRS1, respectively. The mean values (± SEM) are shown. * P < 0.05 (Student’s T-test).

We next compared insulin-regulated glucose homeostasis in HFD-fed WT and AnxA6-KO mice. In line with HFD inducing hyperglycemia [58–61], fasting glucose levels were elevated compared to chow-fed conditions (see Fig 1D) and greater than 140 mg/dl in both mouse strains (Fig 4A). HFD-fed WT and AnxA6-KO mice had comparable response to a glucose challenge including similar glucose tolerance and glucose-stimulated release of insulin (Fig 4B). Taken together, these results indicate that glucose-induced insulin production and insulin-dependent blood glucose lowering were unaltered in AnxA6-KO mice.

Upon closer inspection of the data described above, insulin levels in the OGTT were ~ 20% lower in AnxA6-KO mice compared to the controls at t = 15 min (Fig 4B). Although not significant, this corresponded to slightly (~ 10%) lower glucose levels at this time point (t = 15 min in Fig 4A). This indicated that despite smaller amounts of blood insulin levels, HFD-fed AnxA6-KO mice were able to lower blood glucose more efficiently. To further explore this trend of increased insulin action with the loss of AnxA6, we next determined blood glucose levels in HFD-fed WT and AnxA6-KO mice after insulin injection (Fig 4C). Insulin effectively reduced blood glucose levels in both mouse strains after 15 min, and in line with the trends observed above, insulin-induced glucose lowering was slightly more effective in the AnxA6-KO mice (Fig 4C).

Given these tendencies of increased insulin sensitivity in lipoprotein profiles (Fig 3) and glucose homeostasis (Fig 4A, 4B and 4C) of HFD-fed AnxA6-KO animals, we speculated that insulin signaling in HFD-fed AnxA6-KO could be increased to a greater extend upon insulin administration. Therefore, liver homogenates of animals injected with and without insulin were prepared and analyzed by western blotting for phosphorylated and total Akt and S6 kinase, key players in insulin-inducible phosphatidylinositol-4,5-bisphosphate 3-kinase and mTOR signaling pathways (Fig 4D). Insulin only moderately (~1.5–2 –fold) induced S6 (P-S6) and Akt (P-Akt) phosphorylation in HFD-fed WT mice (compare lane 1–2 with lane 3–4, for quantification see Fig 4E and 4F), which is indicative of HFD-induced insulin resistance [56, 58–60]. In contrast, S6 and Akt phosphorylation was increased more strongly (~3–3.5 –fold) in HFD-fed AnxA6-KO mice following insulin injection (compare lane 5–6 with 7–8). Lack of increased IRS1 phosphorylation in WT and AnxA6-KO mice after insulin administration suggested that loss of AnxA6 mainly modulates S6 kinase activity via insulin signaling (Fig 4D, for quantification see Fig 4G). Insulin receptor levels were comparable in both mouse strains (S1B Fig). Hence, the mild improvement of insulin sensitivity in OGTT and insulin tolerance tests (ITT) in HFD-fed AnxA6-KO mice (Fig 4) correlated with increased insulin-inducible signaling of phosphatidylinositol-4,5-bisphosphate 3-kinase and mTOR pathways.

### Pyruvate administration reveals altered gluconeogenesis in AnxA6-KO mice

**Fig 5:**
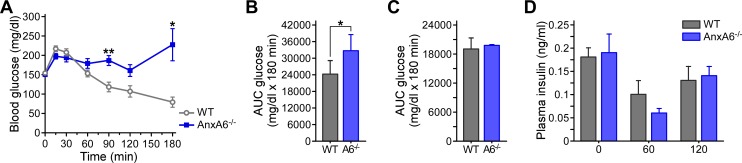
Pyruvate tolerance test (PTT) in HFD-fed WT and AnxA6-KO mice. **(A-B)** HFD-fed and chow fed **(C)** WT and AnxA6-KO (AnxA6^-/-^, A6^-/-^) mice were starved for 4 h prior to intraperitoneal pyruvate administration (2 g/kg body weight). Blood glucose levels were monitored for 0–180 min (mean ± SEM; n = 19 at 0 min, n = 15 at 15, 30, 60 min, n = 10 at 90 min, n = 5 at 120, 180 min); **(B-C)** Area under the curve (AUC, mean ± SEM) of glucose levels during the PTT of **(B)** HFD-fed and **(C)** chow-fed WT and AnxA6-KO mice. **(D)** Plasma insulin levels of HFD-fed WT and AnxA6-KO mice during the PTT at t = 0, 60 and 120 min following pyruvate administration (mean ± SEM; n = 4–5 for each time point). * P < 0.05, ** P < 0.01; * P <0.05 (Student’s T-test).

We next examined hepatic gluconeogenesis, which is often de-regulated upon HFD feeding due to the development of hepatic insulin resistance [66,67]. HFD-fed mice were starved for 4 h, followed by intraperitoneal administration of the gluconeogenic substrate pyruvate. Pyruvate caused a rapid increase of blood glucose levels (t = 15–30 min) in HFD-fed WT animals (Fig 5A). At later time points (t = 60–180 min), insulin-induced suppression of gluconeogenesis led to a rapid decline of blood glucose [66,67]. Similar to the WT mice, pyruvate administration caused an elevation of blood glucose in HFD-fed AnxA6-KO animals within 15–30 min (Fig 5A). However, AnxA6-deficient mice failed to effectively lower blood glucose levels at later time points (60–180 min), and continued to exhibit hyperglycemia. These differences were statistically significant (Fig 5A: 90 min WT 117.70 ± 13.7 mg/dl, AnxA6-KO 186.10 ± 14.2 mg/dl, p = 0.002, n = 10; 180 min WT 78.60 ± 16.4 mg/dl, AnxA6-KO 227.40 ± 52.1 mg/dl, p = 0.016, n = 5; see also area under the curve (AUC) in Fig 5B). Nevertheless, changes in insulin levels 60 and 120 min after pyruvate administration were comparable in HFD-fed WT and AnxA6-KO animals (Fig 5D). Hence, HFD-fed AnxA6-KO mice displayed insulin-induced glucose lowering in OGTT and ITT assays (Fig 4A, 4B and 4C), yet failed to downregulate gluconeogenesis despite normal insulin levels. Together with the insulin signaling response in livers of AnxA6-KO mice (Fig 4D and 4F), this indicated that insulin-stimulated suppression of gluconeogenesis was de-regulated in HFD-fed mice lacking AnxA6.

To examine if this observation in AnxA6-deficient animals was due to HFD feeding, we next compared glucose levels in PTT assays of chow-fed WT and AnxA6-KO mice. Following pyruvate administration, both WT and AnxA6-KO mice initially showed a similar increase of blood glucose levels (30 min WT 125.3 ± 17.4 mg/dl; AnxA6-KO 115.3 ± 10.2 mg/dl; n = 4), followed by a comparable lowering to basal blood glucose levels thereafter (120 min WT 96.8 ± 33.0 mg/dl; AnxA6-KO 97.0 ± 4.4 mg/dl; n = 4). The area under the curve (AUC, 0–180 min) for glucose levels (mean ± SEM) during the PTT of chow-fed WT and AnxA6-KO mice was comparable (WT 19154 ± 2237 mg/dl; AnxA6-KO 19828 ± 143.8 mg/dl) (Fig 5C). Taken together, only HFD-, but not chow-fed AnxA6-KO displayed higher blood glucose levels after pyruvate administration, indicating that insulin-inducible regulatory events responsible for the suppression of gluconeogenesis were compromised in these animals after HFD-induced metabolic stress.

### Pyruvate administration triggers insulin signaling in HFD-fed AnxA6-KO mice

**Fig 6:**
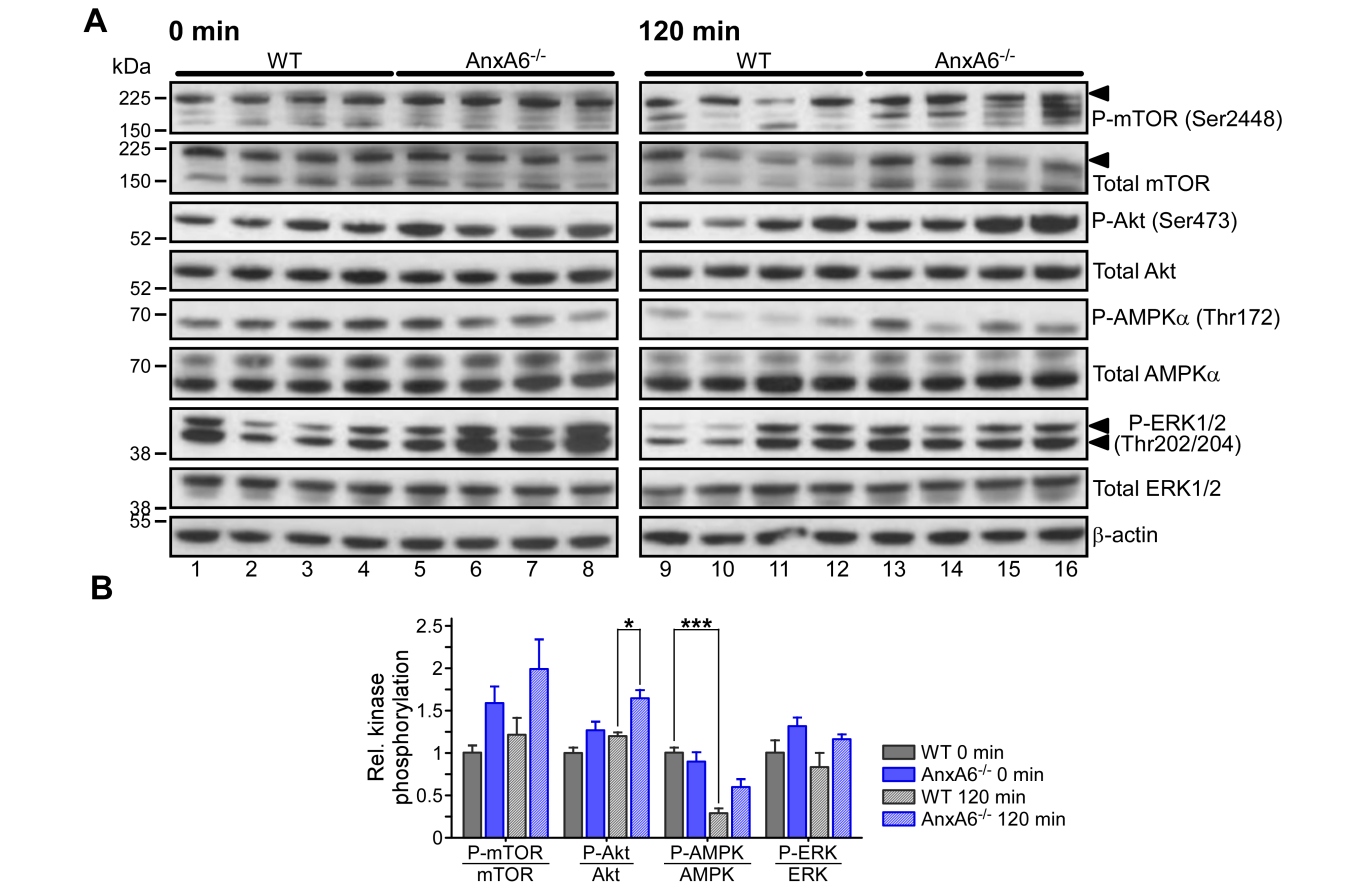
Insulin signaling during the PTT of HFD-fed WT and AnxA6-KO mice. **(A)** Lysates from liver samples from WT and AnxA6-KO (AnxA6^-/-^) mice before (0 min; WT lane 1–4, AnxA6-KO lane 5–8) and 120 min after pyruvate administration (120 min; WT lane 9–12, AnxA6-KO lane 13–16; n = 4 per group) were analyzed by western blotting for phosphorylated/total mTOR (Ser2448), Akt (Ser473), AMPKα (Thr172), and ERK1/2 (Thr202/204). β-actin served as loading control. Molecular weight markers are shown. Black arrowheads point at P-/Total mTOR, respectively. **(B)** Relative levels of activated mTOR, Akt, AMPKα, and ERK1/2 were quantified and normalized to total mTOR, Akt, AMPKα, and ERK1/2, respectively. The mean values (± SEM) relative to WT at 0 min are shown (* P < 0.05, *** P < 0.001 for Student’s T-Test).

Regulation of blood glucose levels upon administration of glucose or insulin in AnxA6-KO animals were comparable to control animals (Fig 4A, 4B and 4C), and correlated with an ability to appropriately activate insulin signaling pathways (Fig 4D and 4F). However, the inability of HFD-fed AnxA6-KO animals to effectively downregulate blood glucose after pyruvate administration, despite normal insulin levels (Fig 5A, 5B and 5D), prompted us to examine the possibility of de-regulated insulin signaling in the liver of AnxA6-deficient animals during the PTT. Therefore, liver extracts from HFD-fed WT and AnxA6-KO animals at t = 0 and 120 min during the PTT were prepared and analyzed by western blotting for mTOR, Akt, AMPKα and Erk1/2 activation (Fig 6A, for quantification see Fig 6B).

Phosphorylation of mTOR and Akt increased by 5–20% in WT and ~ 30% in AnxA6-KO mice over the time course of the PTT, indicating that the elevation of blood glucose levels after pyruvate administration and concomitant insulin response (Fig 5A, 5B and 5D) triggered signaling events in both WT and AnxA6-KO mice (Fig 6A: compare lanes 1–4 with 9–12 for WT and lanes 5–8 with 13–16 for AnxA6-KO, for quantification see Fig 6B). Interestingly, and in line with the increased insulin signaling observed in HFD-fed AnxA6-KO mice upon insulin injection (Fig 4D and 4F), prior (t = 0) and 120 min after pyruvate administration, mTOR as well as Akt phosphorylation was elevated by 5–20% and 25–30% in AnxA6-KO mice compared to controls, respectively.

In addition, phosphorylation of the energy sensor AMPK, which is activated upon shortage of cellular ATP levels [68], was monitored. In agreement with AMPK activation decreasing in energy abundance, phosphorylation of the catabolic subunit AMPKα declined approximately 2–4 -fold during the PTT in both HFD-fed WT and AnxA6-KO animals (compare lanes 1–4 with 9–12 for WT and lanes 5–8 with 13–16 for AnxA6-KO, for quantification see Fig 6B). Phosphorylation of Erk1/2, which can be activated by insulin [69], was also examined. Both HFD-fed WT and AnxA6-KO animals showed a reduction of Erk1/2 phosphorylation during the PTT. In support of AnxA6 inhibiting Erk1/2 activation [11,37–39], and increased insulin signaling in AnxA6-KO mice (Figs 4 and 6), Erk1/2 phosphorylation was elevated in AnxA6-KO mice before and after pyruvate administration.

Collectively, mTOR, Akt, AMPKα and Erk1/2 phosphorylation patterns were comparable in HFD-fed WT and AnxA6-KO animals, most likely reflecting changes in insulin levels during the PTT. Although these observations do not fully rule out differences in mTOR and Akt phosphorylation in chow-fed AnxA6-KO animals, comparable handling of blood glucose levels after glucose, pyruvate and insulin challenge (Figs 1D, 1E and 5C), suggest that AnxA6 depletion does not significantly alter response to insulin signaling in mice on a chow diet. In line with data described above (Fig 4D and 4F), signaling magnitude of insulin-responsive pathways appeared slightly elevated in AnxA6-KO mice. This was not due to upregulated insulin receptor expression, which was comparable in WT and AnxA6-KO animals before and during the PTT (S2 Fig). In respect to mTOR and Akt signaling pathways, mTOR activation can inhibit gluconeogenesis [70], while Akt phosphorylation can decrease expression of gluconeogenic genes [71]. However, both of these mechanisms did not appear to confer blood glucose lowering during the PTT in AnxA6-KO mice, raising the possibility of defective response of targets downstream mTOR and/or Akt in AnxA6-KO animals.

### Expression patterns of transcription factors during PTT of HFD-fed WT and AnxA6-KO mice

**Fig 7:**
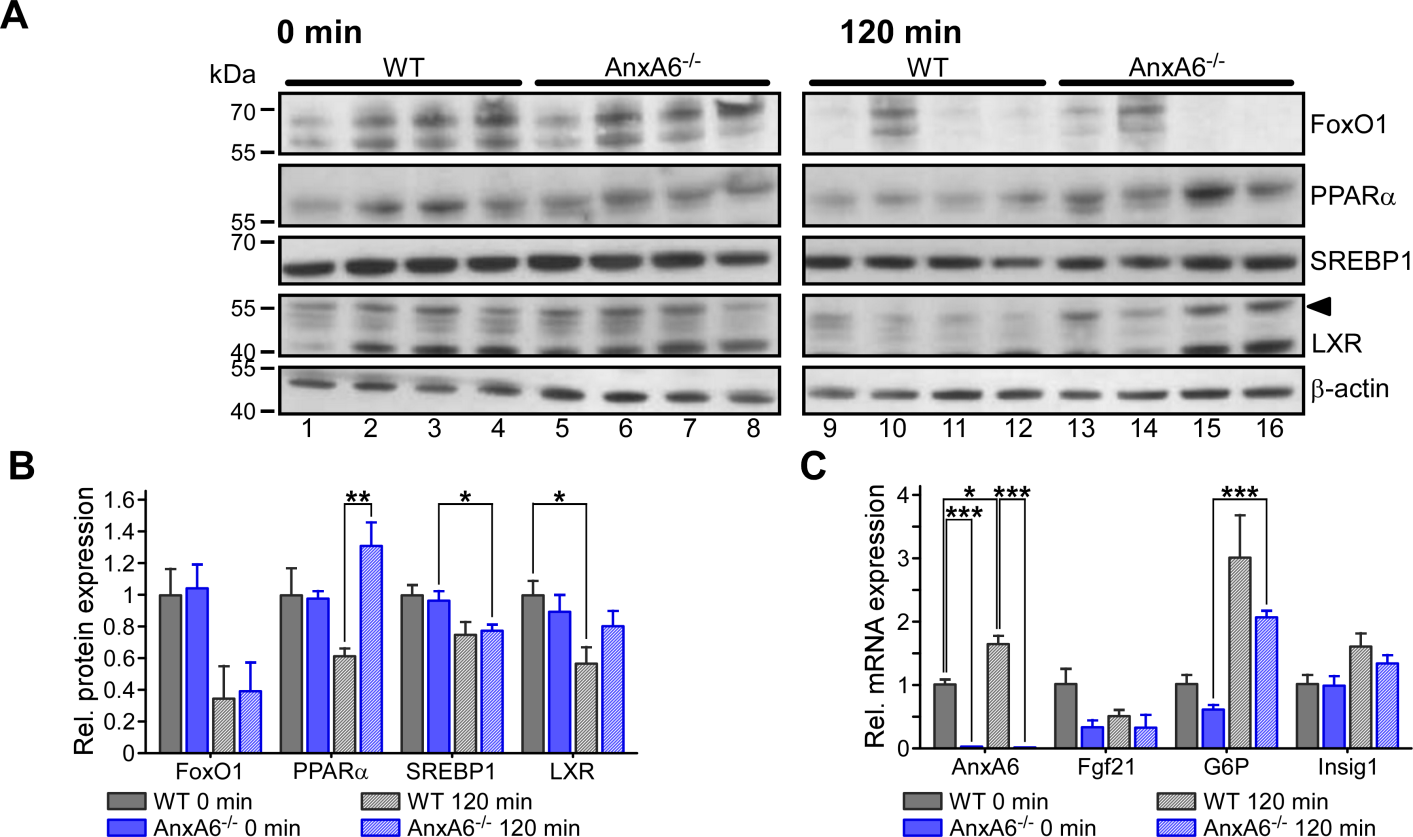
Expression of nuclear transcription factors during the PTT of HFD-fed WT and AnxA6-KO mice. **(A)** Nuclear fractions from liver samples from WT and AnxA6-KO (AnxA6^-/-^) mice before (0 min; WT lane 1–4, AnxA6-KO lane 5–8) and 120 min after pyruvate administration (120 min; WT lane 9–12, AnxA6-KO lane 13–16; n = 4 per group) were prepared and analyzed by western blotting for the transcription factors FoxO1, PPARα, SREBP1 and LXR. β-actin served as loading control. Molecular weight markers are shown. Arrowhead points at LXR. **(B)** Relative levels of FoxO1, PPARα, SREBP1 and LXR were quantified and normalized to β-actin expression. The mean values (± SEM) relative to WT at t = 0 min are shown. **(C)** RNA from HFD-fed WT and AnxA6-KO livers before (0 min) and 120 min after pyruvate administration was isolated (n = 4 per group). cDNA was generated and RT-PCR for AnxA6, fibroblast growth factor 21 (Fgf21), glucose-6 phosphatase (G6P) and insulin induced gene 1 (Insig1) was performed as described in Material and Methods. Relative mRNA expression was normalised to the housekeeper Tbp levels using the ΔΔCT method. The expression relative to the WT at t = 0 min is shown. * P < 0.05, ** P < 0.01, *** P < 0.001 (Student’s T-Test).

In liver, Akt and mTOR signaling regulates several transcription factors that balance expression of genes driving gluconeogenic or glycolytic pathways. These include FoxO1, SREBP1, as well as LXR [71–73], all of which downstream of signal transduction pathways, modulating their ability to regulate genes central to gluconeogenesis, such as phosphoenolpyruvate carboxykinase and G6P. We therefore examined if de-regulated transcription factors downstream of Akt and mTOR and linked to glycemic control could be responsible for the inability of HFD-fed AnxA6-KO mice to downregulate gluconeogenesis. As nuclear translocation is critical for the activation of the abovementioned transcription factors, we prepared nuclear fractions from liver extracts and compared the levels of FoxO1, SREBP1 and LXR during the PTT (Fig 7A and 7B). In both mouse strains, administration of pyruvate led to reduced nuclear amounts of FoxO1, SREBP1 and LXR after 120 min, indicating similar efficacy of insulin signaling pathways to regulate nuclear localization and activity of these transcription factors (Fig 7A and 7B). Indeed, RT-PCR analysis of FoxO1, SREBP1 and LXR target genes G6P and insulin induced gene 1, which are reflective of hepatic insulin response, showed similar changes in their mRNA expression profiles over the course of the PTT in both mouse strains (Fig 7C). Taken together, the hyperglycemia observed in the PPT of HFD-fed AnxA6-KO mice (Fig 5A) was not due to impaired insulin signaling or altered activity of insulin-sensitive transcription factors modulating genes responsible for glycemic control during the PTT.

In addition, we examined nuclear amounts of the transcription factor PPARα, which can also promote gluconeogenesis [74]. Similar to all other transcription factors analyzed during the PTT, nuclear amounts of PPARα in WT mice were strongly reduced 120 min after pyruvate administration (Fig 7B, compare t = 0 and t = 120 min). In strong contrast, nuclear PPARα levels increased by ~30% in AnxA6-KO mice 120 min after pyruvate gavage. However, mRNA expression of the PPARα target gene Fgf21, which is critical for hepatic gluconeogenesis [75], was not elevated under these conditions in AnxA6-KO mice (Fig 7C). Likewise, expression levels of SR-BI and several other established (CPT1α) and potential (GLUT2) PPARα target genes in hepatocytes [74], were comparable in AnxA6-KO and WT mice (S1B and S3 Figs), indicating that despite upregulated PPARα levels, limiting amounts of PPARα co-activators or ligands might determine PPARα target gene expression in AnxA6-KO animals.

Finally, we also monitored AnxA6 levels in WT and AnxA6-KO mice during the PTT (Fig 7C). RT-PCR confirmed the identity of the AnxA6-KO strain, but interestingly, a 50–60% increase of AnxA6 mRNA levels (p <0.05) were observed after pyruvate administration in WT mice. However, despite a potential FOXO1 binding site upstream the AnxA6 gene described recently [76], AnxA6 mRNA and protein levels were not increased in pyruvate or insulin-incubated hepatic HuH7 (S3B and S3C Fig) and HepG2 (data not shown) cell lines, indicating that AnxA6 upregulation in HFD-fed animals during the PTT is complex, possibly required for appropriate metabolic response to downregulate hepatic gluconeogenesis.

### Alterations in liver morphology of HFD-fed AnxA6-KO mice

**Fig 8:**
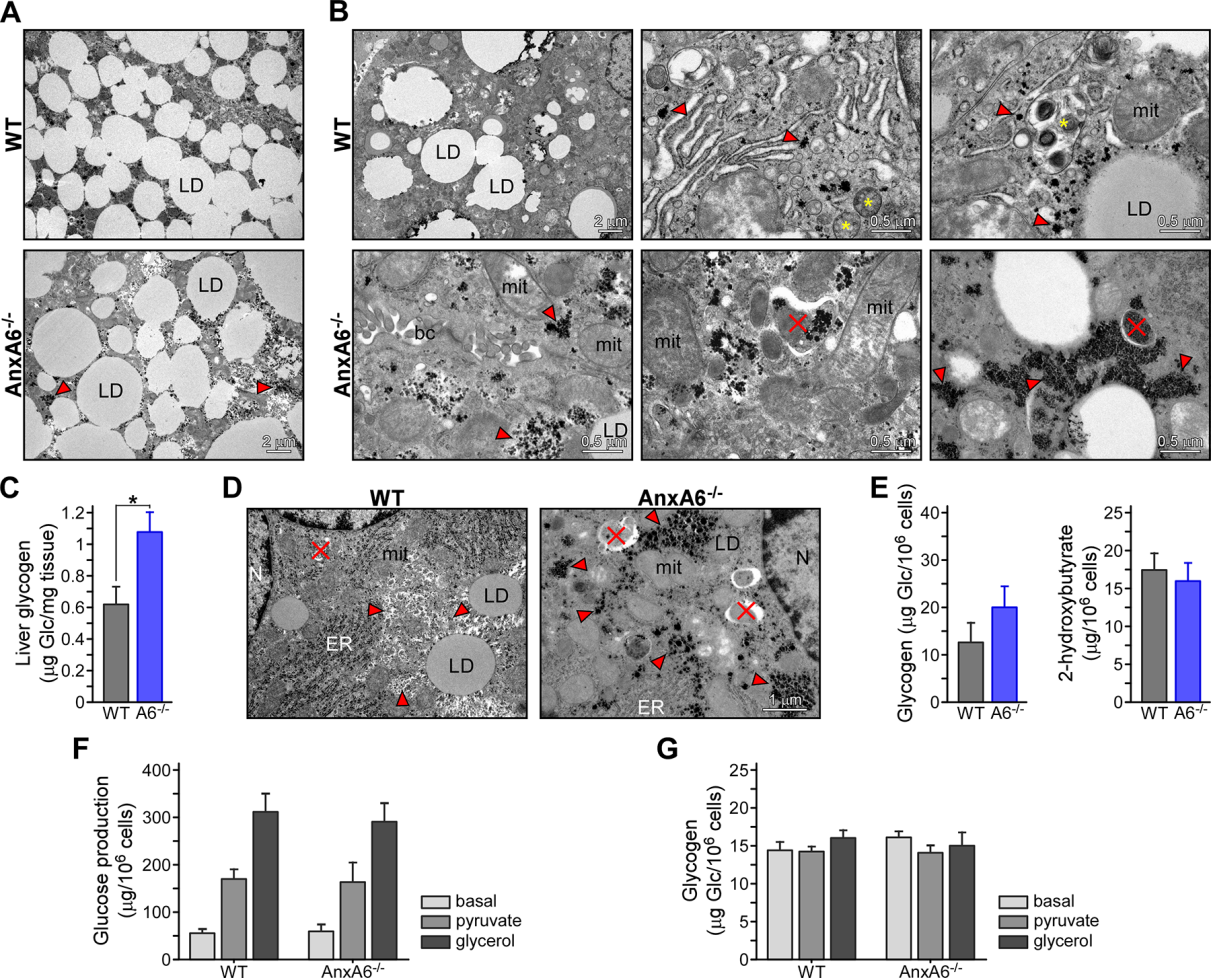
Lipid and glycogen accumulation in HFD-fed AnxA6-KO mice. **(A-B, D)** Liver tissue sections isolated from **(A-B)** HFD-fed and **(D)** chow-fed WT and AnxA6-KO (AnxA6^-/-^) mice were analyzed by electron microscopy. Representative images from 4 animals per group are shown. Bar is 0.5–2 μm as indicated. **(A)** HFD-fed AnxA6-KO mice show fewer, but larger lipid droplets. **(B, D)** Glycogen accumulation (red arrowheads) and glycogen autophagy (red cross) in HFD- and chow-fed AnxA6-KO mice. Bile canaliculi (BC), lipid droplet (LD), mitochondria (mit), endoplasmic reticulum (ER). **(C)** Glycogen content in livers from chow-fed WT and AnxA6-KO mice. **(E)** Primary hepatocytes from chow-fed WT and AnxA6 KO-mice were isolated and lysed to measure glycogen content. The media was collected to determine release of ketone bodies (2-hydoxybutyrate). **(F-G)** Primary hepatocytes from chow-fed WT and AnxA6 KO-mice were isolated, fasted for 6 h without glucose and then incubated with 2 mM pyruvate or 20 mM glycerol for 24 h as indicated. The media was collected and glucose secretion was determined. Cells were lysed and glycogen was extracted (see Methods for details; mean ± SEM; n = 3). * P <0.05 (Student’s T-test).

Given the cross-talk between hepatic lipid and glucose metabolism, and the observation that AnxA6 deficiency influenced the mode how hepatic cells stored excess neutral lipids, leading to reduced LD numbers in livers from AnxA6-KO mice [42], we compared liver morphology by transmission electron microscopy (Fig 8A and 8B). In line with HFD feeding inducing hepatic lipid accumulation, electron micrographs from both mouse strains showed substantial steatosis. However, fewer, but larger LDs were observed in AnxA6-KO livers (Fig 8A), supporting our previous findings that loss of AnxA6 reduces the ability to generate lipid droplets [42]. Interestingly, electron-dense clusters, rosette-like granules of beta particles, representing glycogen stores [77] were detectable in both mouse strains, but were increased in the livers of HFD-fed AnxA6-KO mice (Fig 8B, red arrowheads in lower panel). Furthermore, glycogen autophagy (glycophagy) was observed in AnxA6-KO livers (Fig 8B, red cross), a process which is still poorly understood in adult animals [78], altogether indicating substantial alterations in glycogen metabolism.

These findings, together with the inability to downregulate blood glucose after pyruvate administration (Fig 5A and 5B), indicated an imbalance of pathways regulating hepatic glycogen storage, gluconeogenesis or glycogenolysis. To address if increased glycogen storage in AnxA6-KO livers only occurred after metabolic stress (HFD), we next determined glycogen levels in chow-fed animals. Indeed, hepatic glycogen levels were also elevated by approximately 70% in chow-fed AnxA6-KO mice compared to control animals (WT 0.62 ± 0.27 μg glycogen/mg liver protein, AnxA6-KO 1.08 ± 0.31 μg/mg, n = 6) (Fig 8C). In addition, increased glycogen stores in electron micrographs from chow-fed AnxA6-KO livers (Fig 8D) and an approximately 35–40% increase in glycogen content in primary AnxA6-KO hepatocytes (WT 12.68 ± 4.69 μg/10^6^ cells, AnxA6-KO 20.13 ± 4.94 μg/10^6^ cells, n = 5) (Fig 8E) was observed. Thus, AnxA6 depletion was sufficient to cause increased glycogen storage independent of HFD-induced metabolic stress.

To gain further insight, we next examined gluconeogenesis in chow-fed WT and AnxA6-KO primary hepatocytes that were starved for 6 h and then incubated ± the gluconeogenic substrates pyruvate or glycerol in glucose-free media for 24 h (Fig 8F and 8G). These studies showed a similar capacity of WT and AnxA6-KO primary hepatocytes to synthesize and secrete glucose (Fig 8F). Ketone bodies (2-hydroxybutyrate) were also comparable in WT and AnxA6-KO hepatocytes (Fig 8E), suggesting normal balance between fatty acid oxidation and gluconeogenesis. Likewise, glycogen levels from WT and AnxA6-KO hepatocytes after starvation were similar, indicating that loss of AnxA6 in primary hepatocytes did not cause a major imbalance of gluconeogenesis and glycogenolysis (Fig 8G). In line with comparable mRNA levels of the glucose transporter GLUT2 in HFD-fed AnxA6-KO and WT mice (S3A Fig), glucose uptake in primary hepatocytes from the AnxA6-KO mice and HuH7-A6KD cells were also alike (not shown). Hence, transient differences in the kinetics and metabolic flux in gluconeogenesis or glycogenolysis, or other yet unknown metabolites and pathways may need to be considered as the underlying cause for increased glycogen storage in AnxA6-KO mice.

In further support of altered glycogen storage upon AnxA6 depletion, glycogen levels in AnxA6-depleted HuH7 hepatocytes (HuH7-A6KD) were elevated and remained high compared to controls in the presence of oleic acid, mimicking neutral lipid accumulation after HFD feeding (S4A Fig). On the other hand, starvation of wildtype and AnxA6-depleted HuH7 hepatocytes in serum- and glucose-free media, followed by pyruvate or insulin treatment, showed a similar induction of glycogen production in both cell lines (S4B Fig). Yet, glucose production was slightly reduced in the presence of insulin only in HuH7 control cells, but not AnxA6-depleted HuH7-A6KD cells (S4B and S4C Fig). Hence, AnxA6 deficiency in mice and in human HuH7 hepatocytes may be associated with an uncoupling of insulin signaling from cellular glucose release.

## Discussion

In this study, we identify AnxA6 deficiency in mice to reduce HFD-induced weight gain and adiposity with moderate changes in lipoprotein profiles compared to controls. Furthermore, modestly increased effectiveness of insulin-regulated glucose clearance, as well as elevated magnitude of insulin signaling suggest loss of AnxA6 to provide improved insulin sensitivity. However, despite functional hepatic insulin signaling networks, HFD-fed AnxA6-KO mice show enhanced hepatic gluconeogenesis when challenged with pyruvate, and both HFD- as well as chow-fed AnxA6-KO animals display increased glycogen storage. Hence, AnxA6 is a novel factor contributing to hepatic glucose homeostasis during metabolic stress, modulating gluconeogenesis and glycogen storage, with potential consequences for the systemic control of glucose in health and disease.

Interestingly, HFD feeding caused less weight gain in AnxA6-KO mice, which correlated with reduced white adipose tissue mass and net amount of secreted adipokines. This observation might reflect reduced adipogenesis, since AnxA6-depleted 3T3-L1 pre-adipocytes showed impaired proliferation [28], which would ultimately diminish triglyceride deposition in fat tissue and consequently, reduce whole body weight gain upon HFD feeding. In fact, pre-adipocyte proliferation is increased in obesity [79], and elevated AnxA6 levels in white adipose tissue of obese mice [28] might reflect a requirement for AnxA6 in molecular pathways governing adipose proliferation. However, it should also be noted that in AnxA6-depleted 3T3-L1 adipocytes, we previously identified increased triglyceride storage due to diminished lipolysis [28]. The underlying mechanisms are not fully understood and may involve de-regulated pathways responsible for the activation of hormone-sensitive lipase [28], but AnxA6 depletion in these cell culture studies did not mimic prolonged HFD feeding, nor address the potential of insulin to repress lipolysis. Hence, in 3T3-L1 adipocytes, we speculate that loss of AnxA6 scaffolding functions required for complex assembly responsible for hormone sensitive lipase activation could influence fat deposition.

In line with prolonged HFD feeding inducing hyperlipidemia, total plasma triglycerides and cholesterol were elevated in both WT and AnxA6-KO strains. AnxA6-KO mice showed slightly increased HDL and other cholesterol-rich lipoproteins, while triglyceride-rich lipoproteins were reduced. The accumulation of HDL- or LDL-like lipoproteins in AnxA6-KO mice may reflect a reduced efficacy to internalize and metabolize cholesterol-rich lipoproteins. As insulin improves HDL levels and function [65], yet also has potential to lower VLDL assembly [63], and increase TRL lipolysis in plasma [64], the lipoprotein profiles of AnxA6-KO mice could indicate an increased impact of insulin on lipid metabolism.

Based on OGTT and ITT assays, insulin-regulated glucose homeostasis was comparable in WT and AnxA6-KO mice under normal (chow) and HFD conditions. In fact, AnxA6 deficiency was associated with a trend towards improved insulin sensitivity as judged by increased hepatic insulin signaling and improved glucose clearance despite slightly lower insulin levels. However, in response to a pyruvate challenge, HFD-fed AnxA6-KO animals revealed an inability to downregulate hepatic glucose production. It is important to note that PTT assays are commonly performed after overnight starvation (> 13 h) to ensure complete glycogen depletion [80]. However, given the abnormal glycogen accumulation in livers of HFD-fed AnxA6 KO-mice, prolonged starvation could have resulted in hypoglycemia. Therefore, a standard short starvation protocol (4 h), which is considered suitable to assess insulin action within a physiological context [80] was chosen. Over the time course of this physiological challenge, insulin is thought to suppress gluconeogenesis [70–74]. In spite of this, plasma insulin profiles, hepatic insulin signaling, as well as expression patterns of insulin-regulated transcription factors FoxO1, SREBP1, LXR and PPARα, and their target genes such as G6P, Fgf21, Insig1, GLUT2, or CPT1α, were comparable in WT and AnxA6-KO mice. Future studies should examine these parameters also upon prolonged starvation, but these findings suggest that at least after short starvation glucose synthesis from pyruvate is enhanced in the HFD-fed AnxA6-KO animals, while insulin signaling and glucose uptake from peripheral organs like muscle as well as the liver are normal in AnxA6-KO mice. However, it should also be taken into account that HFD feeding is well known to provoke peripheral as well as hepatic insulin resistance [1–3,57,64]. Small improvements in insulin signaling in the liver of AnxA6-KO mice might be masked by altered peripheral insulin sensitivity, which results in only small changes affecting overall glucose tolerance and insulin sensitivity.

Alternatively, defects unrelated to insulin signaling could be responsible for the continuous glucose production after pyruvate challenge in HFD-fed AnxA6-KO mice. In fact, increased hepatic glycogen storage was observed. Interestingly, this was not restricted to HFD-fed animals, but was also observed in chow-fed AnxA6-KO animals, indicating that AnxA6 depletion, even in the absence of metabolic stress (HFD), causes increased hepatic glycogen storage. The underlying mechanism remains to be elucidated, as loss of AnxA6 did not significantly alter gluconeogenesis, ketone bodies, or glycogenolysis in primary hepatocytes. In addition, we previously identified AnxA6 depletion in differentiated 3T3 adipocytes to reduce lipolysis [28]. Together with comparable levels of systemic triglycerides and free fatty acids in WT and AnxA6-KO animals, it appears unlikely that increased fatty acid flux from adipose tissue could serve as metabolite for glycogen synthesis in the AnxA6-KO liver. Hence, we speculate that transient differences in the kinetics and metabolic flux in hepatic gluconeogenesis or glycogenolysis, or yet unknown other metabolites feeding into gluconeogenesis could be responsible for elevated glycogen storage in AnxA6-KO animals.

Electron micrographs identified glycogen autophagy in HFD- and chow-fed AnxA6-KO livers. Hepatic glycogen autophagy is normally upregulated only upon high glucose demand and involves glycogen sequestration into autophagosomes, and subsequent degradation by autolysosomes. Hence, increased glycogenolysis and/or glycophagy may contribute to the inability of HFD-fed AnxA6-KO mice to downregulate hepatic glucose output after a pyruvate challenge. Interestingly, Ca^2+^ influx into lysosomes to increase acid glucosidase activity and promote glycogen degradation is critical for glycogen autophagy [77]. In Type II glycogen storage disease (Pompe’s disease), lack of lysosomal acid glucosidase leads to the accumulation of glycogen within autophagic vesicles [77]. Given the profound defects in Ca^2+^ homeostasis in Pompe’s disease [81], these observations might point at Ca^2+^-related defects in AnxA6-KO mice.

The underlying mechanism how AnxA6 deficiency impacts on hepatic glucose homeostasis still has to be identified. Although AnxA6-KO mice were originally considered to lack a prominent phenotype [47], defects related to the fundamental themes in annexin biology, Ca^2+^ signaling and membrane function, have emerged. Cardiomyocytes from AnxA6-deficient mice displayed higher contractility and accelerated removal of diastolic Ca^2+^ from the cytoplasm [82]. AnxA6-KO chondrocytes exhibited delayed terminal differentiation, most likely due to an inability to raise intracellular Ca^2+^ levels [83]. Also, several cell types of the AnxA6-KO mice showed abnormal mitochondrial morphology, decreased mitochondrial Ca^2+^ uptake and increased cytosolic Ca^2+^ transients [84]. One could envisage that this impaired mitochondrial respiration may cause a reduced flux of pyruvate through the tricarboxylic cycle, thereby increasing the availability of pyruvate for hepatic glucose synthesis. In fact, mice lacking phosphatidylethanolamine N-methyltransferase have normal insulin sensitivity, modestly ameliorated glucose tolerance and highly improved pyruvate tolerance [85]. In these mice, it was suggested that higher ATP production from pyruvate carboxylation could prevent hyperglycemia.

In addition, examining physiological challenges identified defective biological responses in AnxA6-deficient animals. Comparing knee joints after interleukin 1β injection or partial meniscectomy, AnxA6-KO mice were characterized by a markedly reduced knee cartilage destruction [86]. We recently identified impaired interleukin-2 signaling in AnxA6-KO mice after an immune challenge [50]. This coincided with alterations in membrane order and distribution of proteins at the plasma membrane in several AnxA6-deficient cell types [50, 87]. Taken together, this may point at AnxA6 deficiency to trigger Ca^2+^-dependent phenotypes that require its scaffolding function to organize membrane domains, signaling platforms and the formation of complex protein networks [9–11,30,37–39].

Many events in hepatic lipid and glucose metabolism are highly coordinated processes and controlled by various mechanisms, including Ca^2+^ homeostasis and coordinated membrane trafficking. Deficiency of the Ca^2+^- and membrane binding protein AnxA6 is shown here to affect hepatic lipid droplet morphology, glucose production and glycogen storage. Future experiments, possibly through the generation of liver-specific AnxA6-KO mouse models, could provide opportunities to identify the molecular players that are affected in hepatic lipid and glucose metabolism in an AnxA6-dependent manner.

## Supporting information

S1 Fig Hepatic lipoprotein and insulin receptor expression in HFD-fed WT and AnxA6-KO mice. **(A)** Western blot analysis of AnxA6 and AnxA2 in crude liver extracts from WT and AnxA6-KO (AnxA6^-/-^) mice (n = 2 per group). **(B)** WT and AnxA6-KO animals (2 per group) were fasted for 4 h to receive insulin (1.5 U/kg bodyweight) (+) or saline (-) via intraperitoneal injection. Livers were removed and membrane fractions were analyzed by western blotting for LDL receptor (LDLR), scavenger receptor B1 (SR-B1), apolipoprotein E (ApoE) and insulin receptor β chain (IRβ). β-actin served as loading control. **(C)** Hepatic mRNA expression of AnxA6, fatty acid synthase (FASN), LDLR and glucose-6-phosphatase (G6P). RNA from HFD-fed WT and AnxA6-KO livers (n = 4 per group) was isolated, cDNA was generated and RT-PCR was performed as described in Material and Methods. Relative mRNA expression was normalized to the housekeeper Tbp using the ΔΔCT method.
(TIF)

S2 Fig Expression of insulin and lipoprotein receptors during the PTT of HFD-fed WT and AnxA6-KO mice. **(A)** Membrane fractions from liver samples of WT and AnxA6-KO (AnxA6^-/-^) mice before (0 min; WT lane 1–4, AnxA6-KO lane 5–8) and 120 min after pyruvate administration (120 min; WT lane 9–12, AnxA6-KO lane 13–16) were analyzed by western blotting for insulin receptor β chain (IRβ), LDL receptor (LDLR), LDL-receptor related protein 1 (LRP-1), apolipoprotein E (ApoE). β-actin served as loading control. Molecular weight markers are shown. Arrowheads point at the mature form of IRβ (red), and IR precursor (black). **(B)** Relative levels of IRβ, LDLR, LRP-1 and ApoE were quantified and normalized to β-actin expression. The mean values (± SEM) relative to WT at t = 0 min are shown. * P < 0.05, ** P < 0.01, *** P < 0.001 (Student’s T-Test).
(TIF)

S3 Fig Relative mRNA expression of AnxA6, GLUT2 and PPARα-responsive genes during the PTT of HFD-fed WT and AnxA6-KO mice. **(A)** RNA from HFD-fed WT and AnxA6-KO (AnxA6^-/-^) livers before (0 min) and 120 min after pyruvate administration was isolated (n = 4 per group). cDNA was generated and RT-PCR for AnxA6, GLUT2, pyruvate dehydrogenase lipoamide kinase isozyme 4 (PDK4), glucose-6 phosphatase (G6P), hydroxyacyl-CoA dehydrogenase α and β (Hadha-a, Hadha-b) and carnitine palmitoyltransferase 1A (CPT1α) was performed as described in Material and Methods. Relative mRNA expression was normalised to the housekeeper Tbp using the ΔΔCT method. The expression relative to the WT at t = 0 min is shown. * P <0.05, ** P < 0.01, *** P <0.001 (Student’s T-test). **(B-C)** HuH7 hepatocytes were starved for 6 h, and incubated ± 2 mM pyruvate and 100 nM insulin for 120 min as indicated. **(B)** RNA was isolated and analyzed by RT-PCR for the expression of AnxA1, AnxA6 and PDK4. Their relative mRNA levels normalized to the housekeeper gene 28s rRNA are given. The data (mean ± SD) is representative for two independent experiments with triplicate samples. **(C)** Cells were lysed and samples were analyzed by western blotting for the expression of AnxA6 and glyceraldehyde 3-phosphate dehydrogenase (GAPDH) as indicated. The data is representative for two independent experiments with duplicate samples.
(TIF)

S4 Fig Glycogen and glucose production in HuH7 hepatocytes. **(A)** HuH7-WT and HuH7-A6KD cells were grown in serum-containing media and incubated overnight ± 0.6 mM oleic acid (OA), or **(B-C)** starved for 3–6 h in serum- and glucose-free media, followed by 120 min ± 2 mM pyruvate or 100 nM insulin as indicated. Cells were lysed and glycogen was extracted. Media glucose and glycogen-derived glucosyl levels were quantified as described (see Methods for details; mean ± SEM; n = 3). * P <0.05, ** P <0.01 (Student’s T-test).
(TIF)

## Abbreviations

Akt: protein kinase B
AMPK: 5' adenosine monophosphate-activated protein kinase
Anx: annexin
Ca^2+^: calcium
CPT1α: carnitine palmitoyltransferase 1A
Erk: extracellular signal-regulated kinase
FoxO1: Forkhead box O1
G6P: glucose-6-phosphatase
GLUT: glucose transporter
HDL: high density lipoprotein
HFD: high fat diet; Insig1, insulin induced gene 1
IRS1: insulin receptor substrate 1
ITT: insulin tolerance test
KO: knockout
LDL: low density lipoprotein
LXR: liver X receptor
mTOR: mechanistic target of rapamycin
OGTT: oral glucose tolerance test
PPAR: peroxisome proliferator-activated receptor
PTT: pyruvate tolerance test
RT-PCR: real-time polymerase chain reaction
S6: ribosomal protein S6 kinase
SR-BI: scavenger receptor BI
SREBP1: sterol regulatory element-binding protein 1
TRL: triglyceride-rich lipoprotein
WT: wildtype
