# Supplementary figures and images for: Altered hepatic glucose homeostasis in AnxA6-KO mice fed a high-fat diet
Source: PLoS One. 2018 Aug 15;13(8):e0201310. doi: 10.1371/journal.pone.0201310 (PMC6093612; doi:10.1371/journal.pone.0201310)

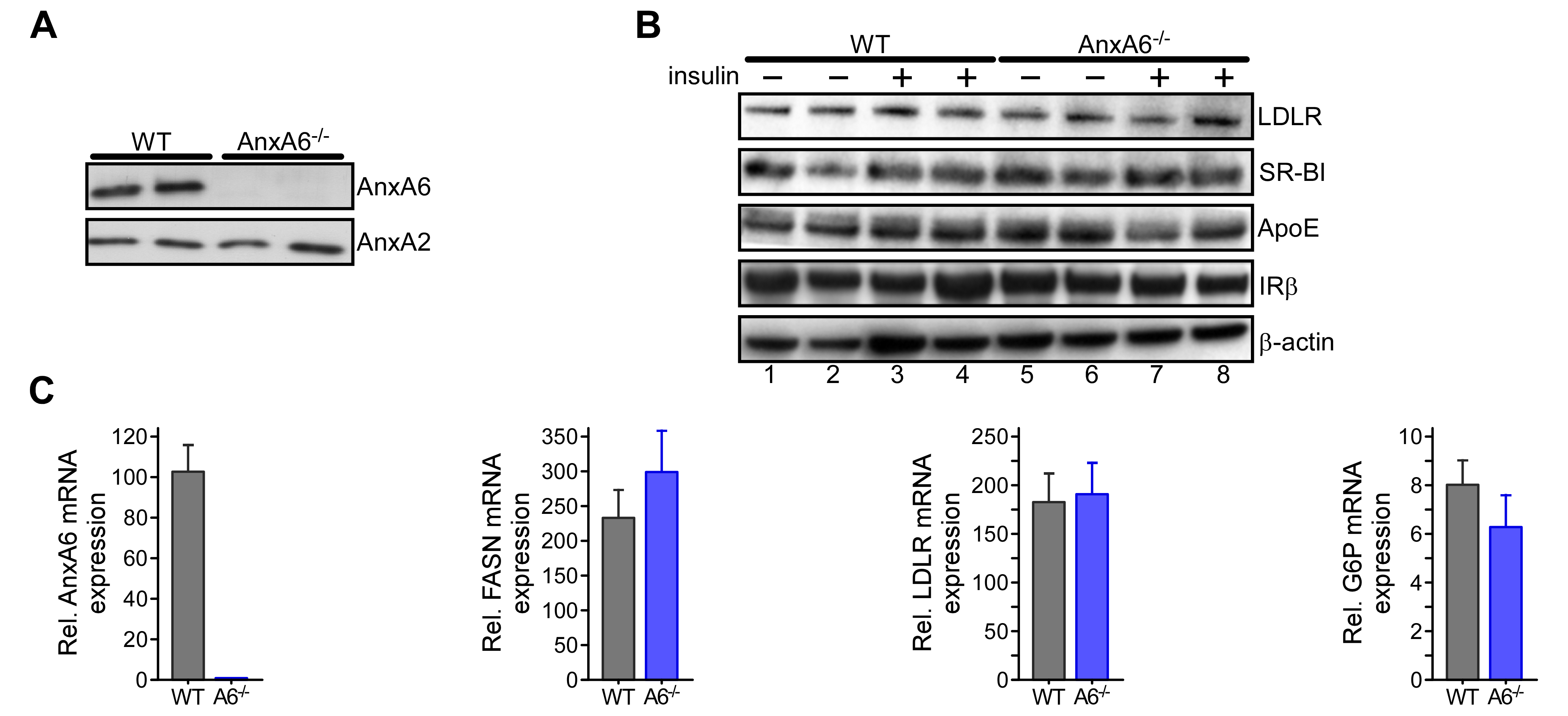

Supplement: S1 Fig — (A) Western blot analysis of AnxA6 and AnxA2 in crude liver extracts from WT and AnxA6-KO (AnxA6-/-) mice (n = 2 per group). (B) WT and AnxA6-KO animals (2 per group) were fasted for 4 h to receive insulin (1.5 U/kg bodyweight) (+) or saline (-) via intraperitoneal injection. Livers were removed and membrane fractions were analyzed by western blotting for LDL receptor (LDLR), scavenger receptor B1 (SR-B1), apolipoprotein E (ApoE) and insulin receptor β chain (IRβ). β-actin served as loading control. (C) Hepatic mRNA expression of AnxA6, fatty acid synthase (FASN), LDLR and glucose-6-phosphatase (G6P). RNA from HFD-fed WT and AnxA6-KO livers (n = 4 per group) was isolated, cDNA was generated and RT-PCR was performed as described in Material and Methods. Relative mRNA expression was normalized to the housekeeper Tbp using the ΔΔCT method. (TIF) [file pone.0201310.s001.tif]

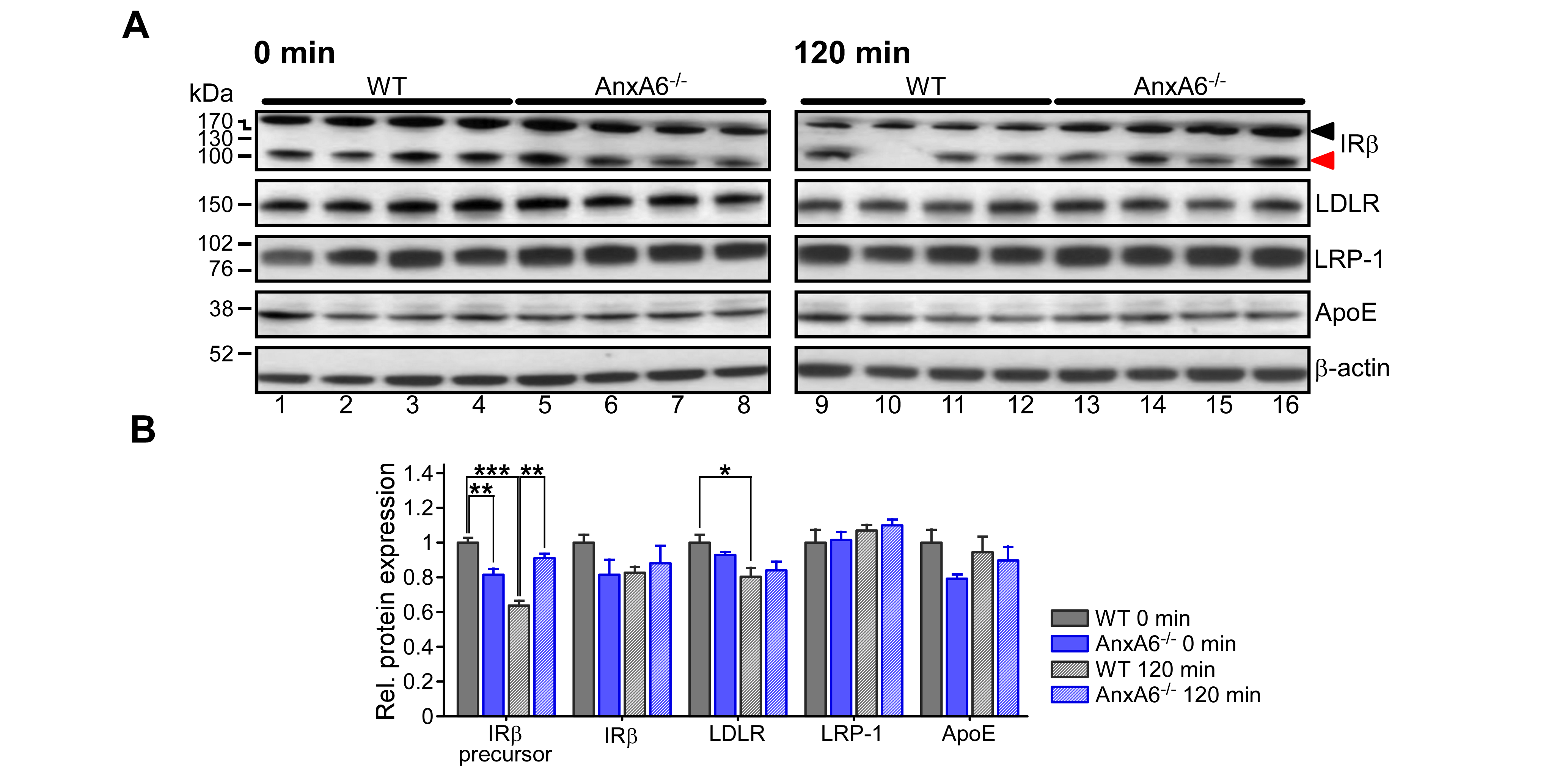

Supplement: S2 Fig — (A) Membrane fractions from liver samples of WT and AnxA6-KO (AnxA6-/-) mice before (0 min; WT lane 1–4, AnxA6-KO lane 5–8) and 120 min after pyruvate administration (120 min; WT lane 9–12, AnxA6-KO lane 13–16) were analyzed by western blotting for insulin receptor β chain (IRβ), LDL receptor (LDLR), LDL-receptor related protein 1 (LRP-1), apolipoprotein E (ApoE). β-actin served as loading control. Molecular weight markers are shown. Arrowheads point at the mature form of IRβ (red), and IR precursor (black). (B) Relative levels of IRβ, LDLR, LRP-1 and ApoE were quantified and normalized to β-actin expression. The mean values (± SEM) relative to WT at t = 0 min are shown. * P < 0.05, ** P < 0.01, *** P < 0.001 (Student’s T-Test). (TIF) [file pone.0201310.s002.tif]

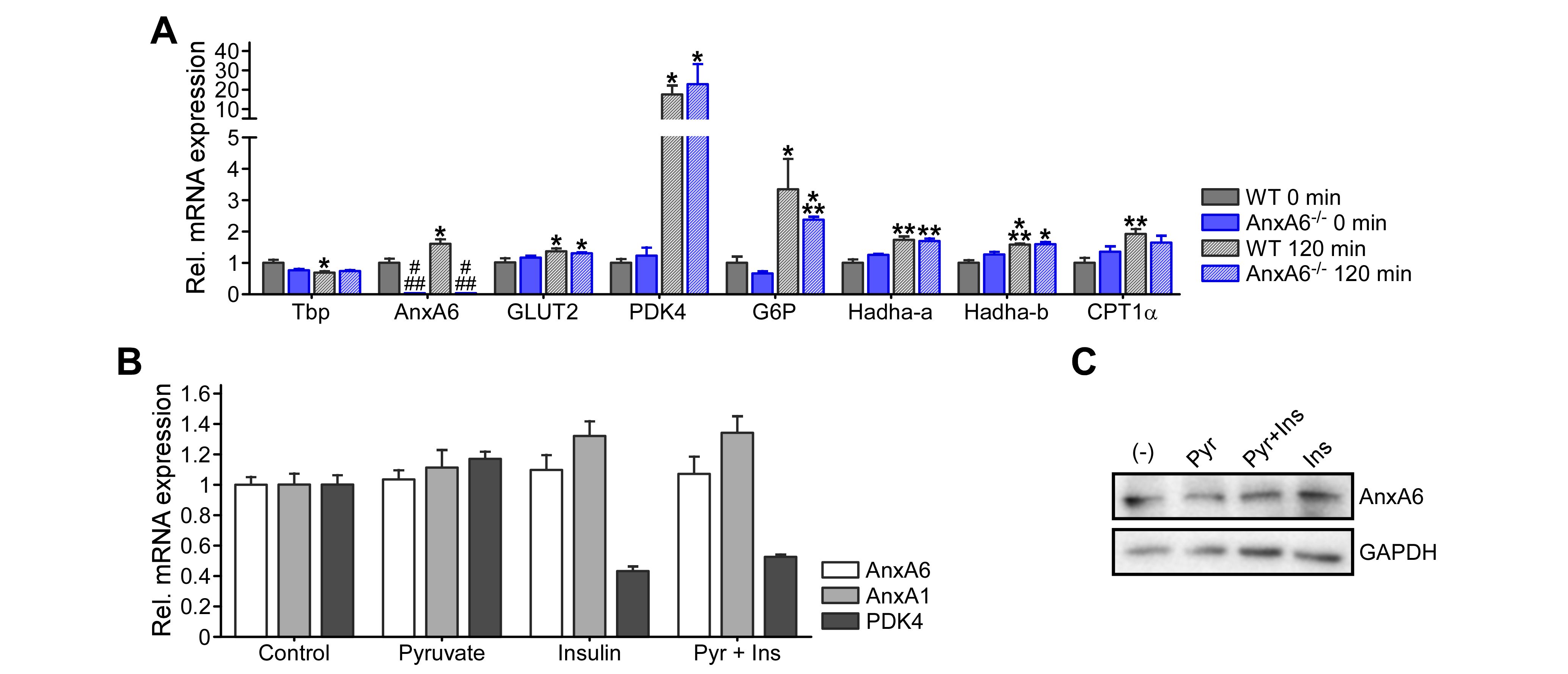

Supplement: S3 Fig — (A) RNA from HFD-fed WT and AnxA6-KO (AnxA6-/-) livers before (0 min) and 120 min after pyruvate administration was isolated (n = 4 per group). cDNA was generated and RT-PCR for AnxA6, GLUT2, pyruvate dehydrogenase lipoamide kinase isozyme 4 (PDK4), glucose-6 phosphatase (G6P), hydroxyacyl-CoA dehydrogenase α and β (Hadha-a, Hadha-b) and carnitine palmitoyltransferase 1A (CPT1α) was performed as described in Material and Methods. Relative mRNA expression was normalised to the housekeeper Tbp using the ΔΔCT method. The expression relative to the WT at t = 0 min is shown. * P <0.05, ** P < 0.01, *** P <0.001 (Student’s T-test). (B-C) HuH7 hepatocytes were starved for 6 h, and incubated ± 2 mM pyruvate and 100 nM insulin for 120 min as indicated. (B) RNA was isolated and analyzed by RT-PCR for the expression of AnxA1, AnxA6 and PDK4. Their relative mRNA levels normalized to the housekeeper gene 28s rRNA are given. The data (mean ± SD) is representative for two independent experiments with triplicate samples. (C) Cells were lysed and samples were analyzed by western blotting for the expression of AnxA6 and glyceraldehyde 3-phosphate dehydrogenase (GAPDH) as indicated. The data is representative for two independent experiments with duplicate samples. (TIF) [file pone.0201310.s003.tif]

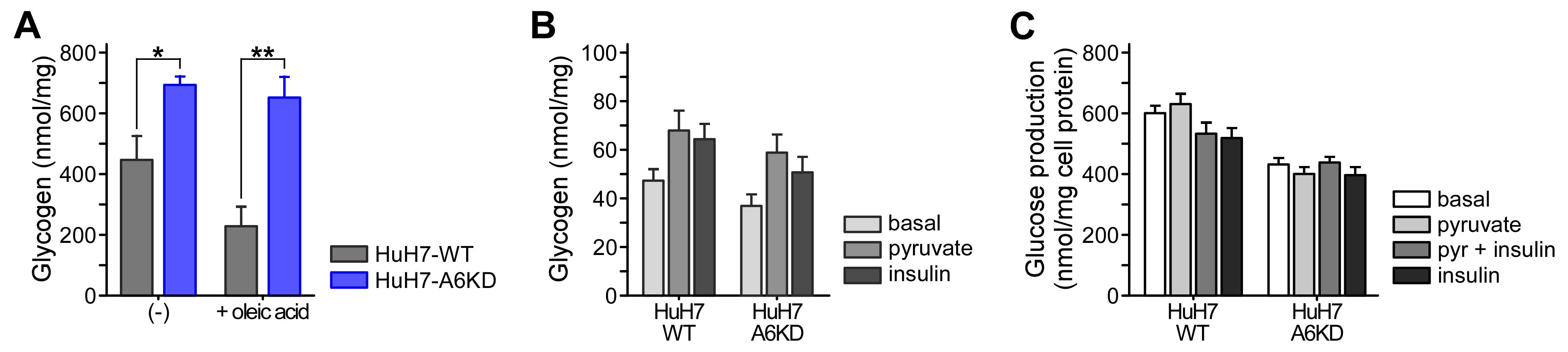

Supplement: S4 Fig — (A) HuH7-WT and HuH7-A6KD cells were grown in serum-containing media and incubated overnight ± 0.6 mM oleic acid (OA), or (B-C) starved for 3–6 h in serum- and glucose-free media, followed by 120 min ± 2 mM pyruvate or 100 nM insulin as indicated. Cells were lysed and glycogen was extracted. Media glucose and glycogen-derived glucosyl levels were quantified as described (see Methods for details; mean ± SEM; n = 3). * P <0.05, ** P <0.01 (Student’s T-test). (TIF) [file pone.0201310.s004.tif]
